# Structural, physicochemical, and gel properties of Wencheng yam (*Dioscorea alata* L.) starch and its application for enhancing set-type yogurt properties

**DOI:** 10.1016/j.fochx.2025.103215

**Published:** 2025-11-07

**Authors:** Ahmed K. Rashwan, Fanrui Zhou, Amged El-Harairy, Wei Chen

**Affiliations:** a Department of Food Science and Nutrition, College of Biosystems Engineering and Food Science, Zhejiang University, Hangzhou 310058, China; b Ningbo Innovation Center, Zhejiang University, Ningbo 315100, China; c Department of Food and Dairy Sciences, Faculty of Agriculture, Qena University, Qena, 83523, Egypt; d College of Materials and Chemical Engineering, Southwest Forestry University, Kunming 650224, China; e Department of Crop and Animal Sciences, Albrecht Daniel Thaer-Institute of Agricultural and Horticultural Sciences, Faculty of Life Sciences, Humboldt-Universität zu Berlin, Albrecht-Thaer-Weg 5, 14195 Berlin, Germany; f Unit of Entomology, Plant Protection Department, Desert Research Center, 1 Mathaf El-Matariya St., El-Matariya, Cairo 11753, Egypt

**Keywords:** Yam starch, Functional properties, Set-type yogurt, Stabilizer material, Physicochemical properties

## Abstract

The purpose of this study was to characterize yam starch (YS) and its effect on set-type yogurt (STY) properties. The YS yield was 13.05 ± 1.22 % and it contained 24.6 ± 1.21 % amylose and 75.4 ± 1.21 % amylopectin, which provides gelling and stabilizing properties to the STY. YS significantly improved water-holding capacity (WHC) and reduced syneresis, with the highest WHC (84.9 %) observed in STY fortified with 1.5 % YS after 21 days of storage at 4 ± 2 °C. Moreover, STY with YS showed better microstructure, whereby less fat globule release occurred. The lactic acid bacteria, such as *Streptococcus thermophilus* and *Lactobacillus bulgaricus*, were viable in each of the treatments over the storage time, where YS maintained their levels close to 7 log CFU/mL. However, sensory evaluation revealed that the 1.0 % YS fortification achieved the highest overall acceptability score, indicating it is the optimal level for balancing improved physicochemical properties with consumer preference. This indicates that YS not only improves yogurt's physicochemical properties but also improves its shelf life, texture, and sensory attributes.

## Highlights

• Greater yam (*Dioscorea alata* L.) is a rich and cheap source for starch.
• Yam starch and its gel showed unique physicochemical and functional properties.
• Yam starch (YS) can be used to supplement set-type yogurt as texture improver.
• YS considered a promising material as stabilizer and replacing costly materials.

## Graphical abstract

**Figure:**
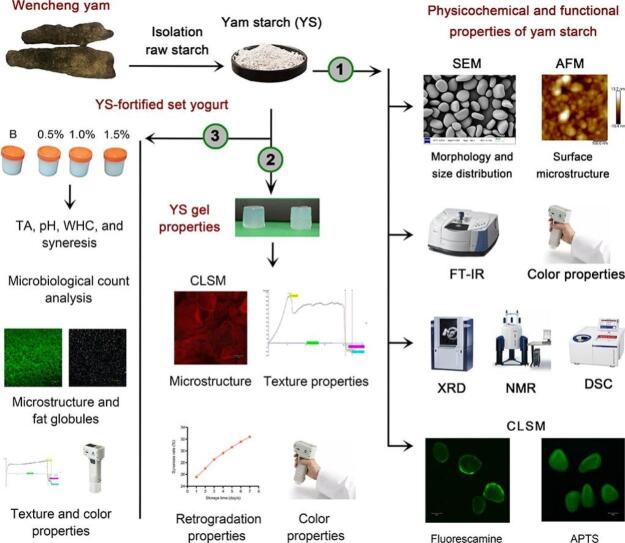

## 1 Introduction

Nowadays, starch plays an important role as a thickener, gelling agent, stabilizer, replacing or extending more costly ingredients, and producing edible coating/film in the food industry. Starches are valued because of their availability, relatively inexpensive cost, and distinctive qualities (Li et al., 2023; Pang et al., 2019). However, using common sources such as potatoes, cereals, and grains to produce starch on a commercial scale becomes one kind of challenge, especially under recent climate changes and the limited food resources accompanying these changes (Rashwan, Younis, et al., 2024). Therefore, using yam tubers as cheap materials to produce starch on a commercial scale for various purposes, including the food industry, pharmaceutical applications, etc., can be a suitable solution to increase the production amount of starch. Yam (*Dioscorea* spp.) is a member of the *Dioscorea* genus and *Dioscoreaceae* family, and there are around 600 *Dioscorea* species identified globally, with over 60 cultivated for food and medicine, with more than 55 found in China (Li et al., 2024; Zou, Xu, Wen, & Yang, 2020). The most often-grown species are *Dioscorea alata* L. (*D. alata* L.) and *Dioscorea polystachya* Turczaninow (*D. polystachya*) (Li et al., 2023; Li et al., 2024).Yams are a cheaper source of starch, especially in Africa and China, where Africa is considered the largest yam tuber producer in the world (86,585 tons), amounting to almost 98 % of the world's yam harvests (FAOSTAT, 2022).

Nevertheless, a few studies investigate the effect of adding yam starch (YS) in food products such as yogurt on the physicochemical and functional properties of these products, especially as a gelling agent, stabilizer, and replacing or extending more costly ingredients (Pérez, Arteaga, Andrade, Durango, & Salcedo, 2021; Tortoe, Akonor, & Ofori, 2019). Yogurts are one of the fermented healthy foods worldwide, which are made by combining pasteurized milk with lactic acid bacteria (LAB), specifically *Streptococcus thermophilus* and *Lactobacillus delbrueckii* subspecies *bulgaricus* under controlled conditions (Rashwan, Younis, et al., 2024). Plain yogurt without stabilizers often has a thinner (Du et al., 2023), watery texture (Sarker, Jung, & Siddiqui, 2023) that can lead to syneresis, where liquid whey separates from the yogurt (Sarker et al., 2023). This separation can make the yogurt less visually appealing and affect its overall mouthfeel and consistency (Du et al., 2023; Fan et al., 2023; Pan, Liu, Luo, & Luo, 2019).

The novelty and necessity of this research are driven by both scientific gaps and pressing industry needs. While previous studies have explored starches from *Dioscorea* species in dairy applications, our study is the first to systematically isolate, comprehensively characterize, and apply native starch from the geographically distinct Wencheng yam (*D. alata* L.) specifically in set-type yogurt. Previous study by Pérez et al. (2021) predominantly focused on stirred-type yogurt with chemically modified starches at low concentrations (0.1–0.5 %). In contrast, our study evaluates a range of native, unmodified starch concentrations (0.5 %, 1.0 %, and 1.5 %) in STY over a commercially relevant 21-day storage period. This distinction is critical because native starches perform differently under varying processing conditions and STY represents a major product category with distinct textural challenges, particularly syneresis. The necessity of this work is driven by three converging factors: the global consumer demand for “clean label” products with natural ingredients; the industrial need for cost-effective, plant-based stabilizers to address persistent quality defects such as whey separation; and the opportunity to valorize regional agricultural resources by transforming Wencheng yam into a high-value functional ingredient. By establishing a clear structure-function relationship through comprehensive starch characterization, this study provides both fundamental scientific insights and practical solutions for the dairy industry, positioning native Wencheng yam starch as a viable, consumer-friendly alternative to modified starches and synthetic stabilizers.

## 2 Materials and methods

### 2.1 Materials and reagents

Fresh Wencheng yams (*D. alata* L.) tubers were collected from Jinshan village, Daxue town, Wencheng County, Wenzhou City, Zhejiang Province, China (27.789602, 120.088783) in November 2023. Liquid milk (Pure milk, Devondale, Australian Dairy (100 mL milk: 3.3 g protein, 3.4 g fat, and 5.1 g carbs)), skimmed milk powder (Anchor™ Trim Milk Powder, New Zealand (100 g milk: protein (26.56 g), fat (0.96 g), and carbs (43.2 g)), and yogurt starter culture DANISCO Yo Mix 883 LYO 50 DCU (containing *Streptococcus thermophilus* and *Lactobacillus delbrueckii* subsp. *bulgaricus*) were purchased from the local supermarket in Hangzhou, Zhejiang Province, China. 8-Aminopyrene-1,3,6-trisulfonic acid trisodium (APTS), Nile red, fluorescamine, and sodium cyanoborohydride were purchased from Sigma-Aldrich (St. Louis, MO, USA). Rhodamine B (Shanghai MaiKun Chemical Co., Ltd., China), Congo red (Dalian Bergolin Biotechnology Co., Ltd., China). All other chemicals and reagents were of analytical grade unless otherwise mentioned.

### 2.2 Raw yam starch preparation

Yam starch was isolated following the method described by Oliveira et al. (2021), with modifications (**Fig. S1**). The yam tubers were submerged in water to remove dirt and cleaned using running water with a sponge. After washing, the tubers were peeled and cut into small pieces (5 cm-thick), submerging the pieces in citric acid solution (10 g/l), and grinding them with drinking water (1:5 *w*/*v*) in a home blender (Midea BL25B31 Blender, China Mainland) for 2 min to obtain a starch slurry. After grinding, the starch solution was filtered through a double- or three-fold cheesecloth to remove the slurry and then sifted through sieves with openings of 500-, 300-, 200-, and 150-mesh (Shaoxing Shangyu Baocheng Instrument Equipment Co., Ltd., China Mainland). The sifted starch was left to stand at 4 °C for 24 h. Subsequently, the decanted starch layer resuspended in pure water and then filtered. The moist yam starch was dried in an air circulation oven (DHG-9023 A, Vollen Lab, China Mainland) at 40 °C for 24 h. Afterward, the starch granules were manually reduced to smaller particles using a mortar and standardized on a 150-mesh sieve.

### 2.3 Yam starch properties

#### 2.3.1 Determination of yield, amylose, and amylopectin contents

The yield of starch extraction was determined by dividing the mass of the final extracted starch by the mass of the yam rhizome used (excluding the peel) and expressing the result as a percentage (Oliveira et al., 2021). Amylose and amylopectin levels were measured through iodine binding spectrophotometry (Shao et al., 2020). To establish a linear correlation between amylose concentration and absorbance, various concentrations of an amylose standard were used. The amylose content in the YS was calculated using these standard curves. Afterward, the amylopectin content was determined by subtracting the amylose content from 100 %, using the formula: amylopectin content (%) = 100 % - amylose content (%).

#### 2.3.2 Physicochemical properties of yam starch

The color indices of samples were determined in terms of the L*, a*, and b* values on the Hunter scale using a colorimeter (Konica Minolta CR-400, Japan). All measurements were performed in triplicates. The L* (lightness to darkness, 100 to 0), a* (redness to greenness, 0 to 100 = red; −80 to 0 = green), and b* (yellowness and blueness, 0 to +70 = yellow; −100 to 0 = blue) as well as hue angle (*H*° = tan^−1^ (b*/a*), chroma (*C* = (a*^2^ + b*^2^) ^½^), and whiteness index (WI = 100 – [(100 - L*)^2^ + a*^2^ + b*^2^]^½^) were reported (Rashwan, Karim, et al., 2024). Bulk density (g/mL) was determined according to Rashwan et al. (2022) by adding 5 g of yam starch into an empty 10 mL graduated cylinder and holding the cylinder on a vortex vibrator for 40 s. The ratio of starch mass and starch volume occupied in the cylinder can determine the bulk density value of starch.

The water solubility (WS), water absorption index (WAI), and swelling power (SP) of yam starch were determined following the methodology proposed by Pérez et al. (2021). In brief, a starch suspension (4 % *w*/*v*) was heated at 80 or 95 °C for 30 min in a water bath, with shaking every 5 min. After cooling to room temperature (25 °C), the suspension was centrifuged at 4000 rpm for 15 min. The supernatant was dried at 105 °C to constant weight (2 h) and the dried residue was cooled in desiccators and weighed to determine the soluble starch content. The water solubility (WS = Ws/W), water absorption index (WAI = Wg/W), and swelling power (SP = (Wg/(W-Ws)), where Wg is the weight of sediment (g), W is the weight of dry solids in the sample (g), and Ws is the weight of dissolved solids in the supernatant (g).

#### 2.3.3 Measurements of morphology and size distribution

The particle shape and surface morphology of YS were examined by scanning electron microscope (ZEISS GeminiSEM 560, Germany) (Shao et al., 2020). A small amount of starch powder was adhered to the carbon double-sided sticky tape fixed on an aluminum stub and put into the ion spray instrument for gold spraying. SEM was used for observation and taking images at three magnifications 200, 500, and 800 X, respectively. The particle sizes of YS were calculated with the scale using the image analysis ImageJ software (NIH, Bethesda, MD, USA) and the obtained data were analyzed using OriginPro 2018 (OriginLab Corporation, Northampton, MA, USA).

#### 2.3.4 Atomic force microscopy (AFM) analysis

The surface microstructure of yam starch was observed using an Atomic Force Microscope (AFM) (Bruker Dimension Icon, Germany) as described by Ma et al. (2021) in the tapping mode. Fifteen milligrams of yam starch were dispersed in 200 μl of deionized water and ultrasonicated for 3 min to ensure even dispersion. Subsequently, 15 μl of the starch suspension was placed on a microscope slide and dried using nitrogen gas. The appropriate testing configuration was selected based on the sample content. The probe (RTESPA-300) was installed and adjusted, and the needle tip was positioned using the Setup view. The sample surface was focused using the Navigate view, followed by lowering the needle. The scanning parameters were then adjusted, and a noise floor image was captured. After the needle was raised, the data were analyzed using the NanoScope Analysis software (Bruker, Bremen, Germany), where the Height Sensor channel was leveled, the image was denoised using the Erase function, and both 2D and 3D profiles were saved.

#### 2.3.5 Fourier transform-infrared spectroscopy (FT-IR) analysis

Absorbance spectra of starch were recorded on a Fourier transform-infrared spectroscopy (FT-IR) spectrometer (NICOLET iS50FT-IR, Thermo Scientific, New York, USA). According to the potassium bromide (KBr) disk method, starch powder was mixed with KBr at a ratio of 1:100 and ground into fine powder, followed by pressing into pellets. The scanning range was from 4000 to 400 cm^−1^ with 64 scans, and the resolution was set at 4 cm^−1^. The spectra were analyzed using Spectrum software, and the data were drawn using OriginPro 2018 (OriginLab Corporation, Northampton, MA, USA) (Rashwan, Karim, et al., 2024).

#### 2.3.6 Determination of crystalline and NMR spectroscopy analysis

The crystallinity patterns are measured by using an X-ray diffractometer (XRD, Bruker D8 Advance, USA) according to Zou et al. (2020) protocol with some slight modifications. It was operated at 40 kV and 40 mA with Cu Kα radiation. The diffraction angle ranged from 5 to 70° with a step size of 0.02 and step time of 0.2 s. The relative crystallinity (%) was calculated using OriginPro 2018 (OriginLab Corporation, Northampton, MA, USA). Furthermore, a nuclear magnetic resonance (NMR) spectroscopy experiment was performed on a JEOL JNM-ECZ500R/M1 spectrometer (11.7 T) operating at a Larmor frequency of 500.16 MHz for ^1^H. These experiments employed a 3.2 mm double-resonance HX MAS solid probe and a spinning frequency of 10.0 kHz. The MAS NMR spectra were recorded using a single-pulse sequence with a π/2 pulse length of 2.06 μs for ^1^H and 2.06 μs for ^13^C (Zou et al., 2020).

#### 2.3.7 Differential scanning calorimetry (DSC)

The thermal properties of the isolated YS were studied using DSC (PerkinElmer DSC 6000, PerkinElmer, USA) following previously published procedures (Shao et al., 2020; Zou et al., 2020). A 5 mg starch powder was mixed with 15 mg of distilled water and then sealed in an aluminum crucible. The mixture equilibrated at 4 °C for 24 h and then at room temperature for 1 h. The crucible was held at 20 °C for 1 min in the DSC furnace and then heated from 20 °C to 110 °C at a rate of 10 °C/min, with test gas N_2_, and flow rate: 50 ml/min (10 % *W*/*V*). The data obtained included the onset temperature (*T*_o_), peak temperature (*T*_p_), conclusion temperature (*T*_c_), the difference between *T*_c_ and *T*_o_ (*ΔT*), and crystal melting enthalpy (*ΔH*).

#### 2.3.8 Staining of starch granules with APTS, fluorescamine, and Congo red

The samples were prepared according to the method described by Ma et al. (2021) for staining of starch granules with 8-Aminopyrene-1,3,6-trisulfonic acid trisodium (APTS) and fluorescamine. A 10 mg starch was stained in 15 μl of APTS solution (10 mM APTS in 15 % acetic acid) and 15 μl of 1 M sodium cyanoborohydride at 30 °C for 15 h, followed by centrifugation at 10,000 ×*g* for 5 min and washing five times with MilliQ water. Separately, starches (15 mg) were dispersed in 0.1 % (*w*/*v*) fluorescamine (0.3 ml) in acetonitrile and 0.15 mL of 0.1 M borate buffer (pH 8.0) at 25 °C for 1 h, then centrifuged at 10,000 ×*g* for 5 min and washed five times with MilliQ water. The samples were suspended in 0.5 ml 50 % glycerol and then observed using a confocal laser scanning microscope (CLSM, ZEISS Introduces LSM 880 with Airyscan, Oberkochen, Germany). For samples stained with APTS, the imaging was collected with scaling X (0.415 μm), scaling Y (0.415 μm), imaging size (x: 1024, y: 1024, channels: 2, 8-bit), dimensions (x: 424.68 μm, Y: 424.68 μm), scan mode (plane), zoom (1.0 or 2.5), objective (Plan-Apochromat 20×/0.8 M27), pixel dwell (2.06 μs), average (1), master gain (Ch2: 640; ChD: 270), digital gain (1.00), digital offset (0.00), pinhole (89 μm), filters (504–568), beam splitters (MBS: MBS 488; MBS_InVis: MBS -405; DBS1: Mirror; FW1: NoneLSM), and lasers (488 nm: 2.0 %). For fluorescamine-stained samples, the imaging was collected with scaling X (0.415 μm), scaling Y (0.415 μm), imaging size (x: 1024, y: 1024, channels: 2, 8-bit), dimensions (x: 424.68 μm, Y: 424.68 μm), scan mode (plane), zoom (1.0), objective (Plan-Apochromat 20×/0.8 M27), pixel dwell (2.05 μs), average (line sum 2), master gain (180), digital gain (1.00), digital offset (0.00), pinhole (114 μm), filters (410–464), beam splitters (MBS: MBS 488; MBS_InVis: MBS -405; FW1: NoneLSM; DBS1: Mirror), and lasers (Track1 (488 nm: 14.0 %); Track2 (405 nm: 15.0 %)).

Moreover, the degree of gelatinization (DG) of YS was determined using a Congo red dye (0.2 %) as a stain (Sorour, Mehanni, Taha, & Rashwan, 2019). An aqueous solution of starch (1 %) was mixed with dye and then heated in a water bath, and samples were taken at 1 °C intervals from 30 °C onwards until the gelatinization temperature was reached. Starch granules were stained at the initial (2.0 % stained), midpoint (50 % stained), and final (98 % stained) stages. The pictures were taken using a fluorescence microscope (Nikon Eclipse Tisingle bondS fluorescence microscope, Tokyo, Japan).

#### 2.3.9 Yam starch gel properties

##### 2.3.9.1 Texture analysis

The texture analysis of yam starch gels (YSG) was carried out in two starch concentrations (6 and 10 % (*w*/*v*)), where starch suspensions were heated at 95 °C for 30 min in a water bath, with shaking every 5 min. After cooling to room temperature (25 °C), the suspensions were kept at 4 °C for 24 h to obtain gels (Ma, Wen, et al., 2022). Texture analysis was performed on starch gel to determine their texture properties using a texture analyzer (TA.XTplusC Texture Analyzer (Stable MicroSystems Ltd., Surrey, UK) equipped with a 30 kg load cell. Using the following settings: pre-test speed 1.0 mm/s (s), post-test speed 10.0 mm/s, test speed 1.0 mm, with minimum triggering force 5.0 g, and test distance 20.0 mm with probe model P/05. All tests were performed at ambient temperature, with the product temperature maintained near 4 ± 2 °C. The tests were conducted on samples prepared in 90 ml Nalgene containers (Nantong Baifu Biotechnology Co., Ltd., China) with dimensions of 58 mm in diameter and 52 mm in height. The samples were taken directly from the refrigerator, maintained at 4 ± 2 °C, and placed on the instrument stage for measurements. The following parameters were calculated from the Exponent software: firmness (g) (maximum force developed within penetration), consistency (g*s) (total positive area), cohesiveness (g) (maximum adhesive force), and viscosity index (g*s) (total negative area).

##### 2.3.9.2 Microstructure analysis

The microstructure of YSG was carried out using a Congo red dye (0.2 %) as a stain. An aqueous solution of starch (3 %) was divided into two glass beakers mixed with the dye and then heated in a water bath. The first one was heated to 85 °C and the second was heated to 95 °C for 30 min, then both cooled down to room temperature. After that, the microstructure of YSG was observed using a confocal laser scanning microscope (CLSM, ZEISS Introduces LSM 880 with Airyscan, Oberkochen, Germany).

##### 2.3.9.3 Freeze-thaw stability

The freeze-thaw stability of starch gels was assessed following the procedure outlined by Huang et al. (2024) with negligible modifications. Briefly, a 10 % starch suspension was prepared and heated in a boiling water bath for 30 min to produce a starch paste. From this, 30 g of paste was placed into a 50 ml centrifuge tube and frozen at −18 °C for 18 h. The tubes were then thawed at 25 °C for 6 h. Following thawing, the samples were centrifuged at 2000 *g* for 15 min, and the separate water was removed. This process was repeated until the water was released and the number of freeze-thaw cycles used was recorded. The starch syneresis rate (%) was calculated using the following equation:

$$\text{Starch syneresis rate}\;\left(\%\right)=\left(\frac{\text{Weight of water separated}}{\text{Weight of starch paste}}\right)\times 100$$

##### 2.3.9.4 Retrogradation properties

Moreover, retrogradation properties were determined as syneresis rate (%) of YSG according to Singh, McCarthy, and Singh (2006) method with some modifications. Starch suspensions (2 %, *w*/*v*) were heated at 90 °C for 30 min in a temperature-controlled water bath with constant stirring. After obtaining the gel, about 20 g starch gel was transferred to a 50 ml tube and then cooled at room temperature (in 6 min) using an ice water bath. Afterward, the YSGs were stored for 7 days at 4 °C, where the syneresis was measured at 1, 2, 3, 4, 5, 6, and 7 days and each day was estimated using three replicates. The exuded water at the end of each cycle was gravimetrically determined, syneresis was calculated as the amount of water exuded from the starch gels expressed as a percentage of the total weight of the sample used.

### 2.4 Preparation of set-type yogurt fortified with yam starch

The yogurt was prepared as described by Rashwan, Karim, et al. (2024) with minor modifications. Briefly, 8 l of commercial homogenized and pasteurized milk were fortified with 1 % (*w*/w) skimmed milk powder and then mixed thoroughly. Then, milk was divided into 4 different groups to obtain STY-control (one group without starch) and three groups fortified with starch including 0.5, 1.0, and 1.5 % starch, and mixed well. Then the mixture was pasteurized at 85 °C for 25 min, cooled down to 42 °C, and then starter culture (40 mg/1 kg milk) was added. The samples were distributed to sterile 100 mL plastic containers (Nalgene containers (Nantong Baifu Biotechnology Co., Ltd., China) with dimensions of 58 mm in diameter and 52 mm in height) to allow easy collection of samples for analysis. Fermentation was carried out at 43 °C for 6 h to develop proper acidity. Then, all STY samples were kept in the refrigerator (4 ± 2 °C) for further analysis after 1st-day, 7th-day, 14th-day, and 21st-day intervals, respectively.

#### 2.4.1 Color analysis, pH, and titratable acidity

The color indices of samples were determined in terms of the L*, a*, and b* values on the Hunter scale using a colorimeter (Konica Minolta CR-400, Japan). All measurements were performed in triplicates. The L* (lightness to darkness, 100 to 0), a* (redness to greenness, 0 to 100 = red; −80 to 0 = green), and b* (yellowness and blueness, 0 to +70 = yellow; −100 to 0 = blue) as well as hue angle (*H*° = tan^−1^ (b*/a*), chroma (*C* = (a*^2^ + b*^2^) ^½^), and whiteness index (WI = 100 – [(100 - L*)^2^ + a*^2^ + b*^2^]^½^) were reported (Rashwan, Karim, et al., 2024). The color difference (*ΔE*) was calculated according to the following equation (Pérez et al., 2021).

$$\mathrm{\Delta E}=\sqrt{{\left(L_{m}-L_{c}\right)}^{2}+{\left(a_{m}-a_{c}\right)}^{2}+{\left(b_{m}-b_{c}\right)}^{2}}$$

Where the subscript “m” stands for the sample of yogurt formulated with yam starch, and “c” stands for the control sample.

A 10-g sample was measured and subsequently diluted with 10 ml of distilled water (previously boiled). The pH of all samples was then assessed using a calibrated pH meter (Apera Instruments, China). Titratable acidity (TA) was determined through titration with 0.1 N NaOH until reaching the phenolphthalein endpoint, and the result was expressed as a percentage of lactic acid (%TA) (Rashwan et al., 2022). TA was calculated using the following equation:

$$\%\mathrm{TA}=\left(\frac{\left(N_{\mathrm{NaOH}}\times V_{\mathrm{NaOH}}\left(\mathrm{mL}\right)\times 0.09\right)}{W_{\mathrm{Sample}}\;\left(g\right)}\right)\times 100$$

Where the milliequivalent weight of lactic acid is 0.09.

#### 2.4.2 Water holding capacity (WHC) and syneresis

Water holding capacity (WHC) and syneresis were estimated according to the methods described by Rashwan, Karim, et al. (2024) , with slight modifications. Briefly, 20 g of the STY samples were centrifuged at 3000 *g* for 15 min at 4 °C. The supernatants were removed within 5 min, and the precipitates were weighed. The WHC and syneresis values were calculated as follows (W = weight):

$$\mathrm{WHC}\;\left(\%\right)=\frac{W_{\text{precipitate}}}{W_{\text{total yogurt sample}}}\times 100$$

$$\text{Syneresis}\;\left(\%\right)=\frac{W_{\text{supernatant}}}{W_{\text{total yogurt sample}}}\times 100$$

#### 2.4.3 Texture analysis of STY

Texture analysis was performed on yogurt to determine its texture properties using a texture analyzer. The following parameters were calculated from the Exponent software: firmness (g) (maximum force developed within penetration), consistency (g*s) (total positive area), cohesiveness (g) (maximum adhesive force), viscosity index (g*s) (total negative area), and gumminess (firmness × cohesiveness). The methodology details can be found in section 2.3.9.1.

#### 2.4.4 Fat globules and microstructure analysis

Fat globules of STY samples were observed after the 7th day of cold storage using a confocal laser scanning microscope (CLSM, ZEISS Introduces LSM 880 with Airyscan, Oberkochen, Germany). The samples were prepared according to the method described by Rashwan, Karim, et al. (2024) with some modifications. Nile red was used to stain fat globules in the STY-supernatant, which was collected in the WHC test. At first, supernatants were diluted five times with deionized (DI) water. Then, Nile red was dissolved in acetone at a concentration of 0.5 mg/ml. Afterward, 20 μL of Nile red solution was mixed with 2 ml diluted STY-supernatant. The mixture was gently shaken and incubated at 25 °C for 10 min in the dark. After that, fat globules contained in the mixture were observed using CLSM. The work conditions, the imaging was collected with scaling X (0.132 μm), scaling Y (0.132 μm), imaging size (x: 1024, y: 1024, channels: 2, 8-bit), dimensions (x: 135.04 μm, Y: 134.82 μm), scan mode (plane), zoom (1.0), objective (Plan-Apochromat 63×/1.4 Oil DIC M27), pixel dwell (2.06 μs), average (1), master gain (Ch2: 630; ChD: 230), digital gain (1.00), digital offset (Ch2: −1.00; ChD: 0.00), pinhole (104 μm), filters (563–623), beam splitters (MBS: MBS 488/561; MBS_InVis: Plate; DBS1: Mirror; FW1: NoneLSM), and lasers (561 nm: 15.0 %).

#### 2.4.5 Microstructure of STY fortified with yam starch

The microstructure of STY samples was examined using CLSM. The samples were prepared according to Pang et al. (2019) with some modifications. Briefly, Rhodamine B was dissolved in deionized (DI) water at a concentration of 0.08 % (*w*/*v*). Then, 40 μL of Rhodamine B solution was used to stain the protein network after mixing with 5 ml yogurt. After gently mixing, the mixtures were incubated at 4 °C for 60 min in the dark. Afterward, the sample was taken to a microscopic slide and covered with a coverslip for examination under CLSM. The work conditions, the imaging was collected with scaling X (0.415 μm), scaling Y (0.415 μm), imaging size (x: 1024, y: 1024, channels: 2, 8-bit), dimensions (x: 424.55 μm, Y: 424.68 μm), scan mode (plane), zoom (1.0), objective (Plan-Apochromat 63×/1.4 Oil DIC M27), pixel dwell (2.06 μs), average (line sum 2), master gain (Ch2: 630; ChD: 210), digital gain (1.00), digital offset (Ch2: −1.00; ChD: 0.00), pinhole (92 μm), filters (563–623), beam splitters (MBS: MBS 488/561; MBS_InVis: Plate; DBS1: Mirror; FW1: NoneLSM), and lasers (561 nm: 10.0 %).

#### 2.4.6 Microbiological count analysis

*Streptococcus thermophilus* and *Lactobacillus bulgaricus* were enumerated using selective agar media (Sarker et al., 2023). Briefly, on days 1, 7, 14, and 21 of the yogurt preparation, samples were thoroughly mixed to disrupt the gel structures. From each sample, 1 mL of yogurt stored at 5 °C was aseptically taken and serially diluted (1:10) with 0.1 % sterile peptone water (Becton, Dickinson and Company, Sparks, MD). Aliquots of 100 μL of the diluted samples were plated in duplicate onto Difco De Man, Rogosa, and Sharpe (MRS) agar (BD, Sparks, MD), adjusted to pH 5.2, and M17 agar (BD, Sparks, MD) containing 10 % w/v lactose (BD, Sparks, MD) to enumerate *Lactobacillus delbrueckii* subsp. bulgaricus and *Streptococcus thermophilus*, respectively. The MRS plates were incubated anaerobically for 72 h at 37 °C using GasPak™ EZ Container Systems (BD, Sparks, MD), while the M17 plates were incubated at 42 °C for 72 h. The counts from the duplicates were expressed as logarithms of colony-forming units per mL of yogurt (log CFU/mL). To determine the total viable count of lactic acid bacteria (LAB) in the yogurt samples, the counts of *Lactobacillus delbrueckii* subsp. bulgaricus and *Streptococcus thermophilus* were combined.

#### 2.4.7 Sensory evaluation of STY

The sensory characteristics of STY samples were assessed on the 1st day and 14th day of cold storage as described by Rashwan, Karim, et al. (2024) with minor modifications. To ensure the sensory analysis's realism, 25 members were randomly selected from Zhejiang University, including master's and Ph.D. students (16 males and 9 females, aged 23–35). These randomly chosen panelists evaluated the samples based on the “degree of liking for appearance, taste, smell, structure, acidity (acidic taste), and acceptability” of STY-control and starch-fortified STYs. Each sample was rated using a 9-point hedonic scale (1 = dislike extremely, 2 = dislike very much, 3 = dislike moderately, 4 = dislike slightly, 5 = neither like nor dislike, 6 = like slightly, 7 = like moderately, 8 = like very much, 9 = like extremely). The panelists scored the samples independently without consulting each other.

### 2.5 Statistical analysis

The experimental data were represented as mean values with standard deviation (mean ± SD). Differences between mean values were established by one-way ANOVA followed by Duncan's multiple range test using SPSS (IBM SPSS, V. 22.0, New York, USA) statistics, GraphPad Prism (v 9.0), and OriginPro 2018 (OriginLab Corporation, Northampton, MA, USA) to design the figures. All the analyses were carried out in triplicate. *p* < 0.05 was considered statistically significant.

## 3 Results and discussion

### 3.1 Yield and functional properties of yam starch

**Fig. 1:**
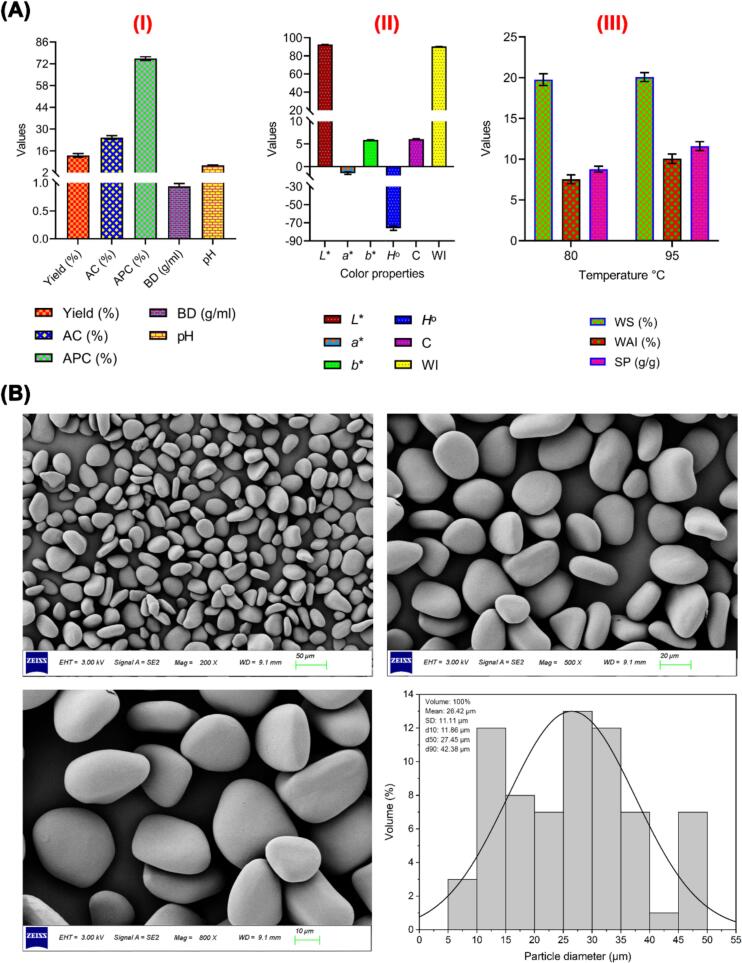
**(A-I)** Yield (%), bulk density (BD g/ml), pH, amylose content (AC %), and amylopectin content (APC %) of yam starch; **(A-II)** Color properties of yam starch (*Dioscorea alata* L.) powder; **(A-III)** Water solubility (WS %), water absorption index (WAI %), and swelling power (SP g/g) of yam starch at 80 and 95 °C. The values are expressed as the mean ± standard deviation (SD), **(B)** The particle shape and surface morphology of yam starch were examined by scanning electron microscope (SEM), and the observation and images were done at three magnifications: 200, 500, and 800×, respectively. The particle sizes of yam starch were calculated with the scale using the image analysis ImageJ software, and the obtained data were analyzed using OriginPro 2018. Here, d (10), d (50), and d (90) are the granule sizes at which 10 %, 50 %, and 90 % of all the granules by volume are smaller. **Here**, L*: Lightness, a*: Redness, b*: Yellowness, *H*°: Hue angle, *C*: Chroma, WI: Whiteness index.

The yield and physicochemical qualities of YS play an important role in determining its appropriateness for diverse applications. The yield of YS varies depending on the yam species and extraction processes, although it normally ranges from 12 to 28 % of the fresh tuber weight (Li et al., 2023; Rashwan, Younis, et al., 2024). Physicochemical features of starch such as bulk density, pH, color properties, water solubility (WS), water absorption index (WAI), swelling power (SP), amylose content, and amylopectin content affect its effectiveness in food and industrial applications, with YS frequently demonstrating high viscosity and gel strength, making it useful for thickening and gelling (Li et al., 2023; Pérez et al., 2021). Fig. 1A-I shows the various properties of YS, including yield (%), amylose (%), amylopectin (%), bulk density (g/ml), and pH, The results showed that the yield of YS reached 13.05 ± 1.22 % (fresh weight (FW)), which indicates a good efficiency of the starch extraction process. Additionally, the YS presented 24.6 ± 1.21 % amylose and 75.40 ± 1.21 % amylopectin, which is important for the functional properties of the starch, including gelatinization and retrogradation properties. Similar results by Oliveira et al. (2021) showed that starch yield extracted from yams using the wet milling method reached 12.34 ± 1.04 % (FW), and YS presented 25.77 % amylose and 74.23 % amylopectin. Besides, the pH value of YS is around neutral (6.8 ± 0.1), indicating the starch is neither too acidic nor too alkaline, which is favorable for most food applications. YS had a high bulk density of 0.94 ± 0.05 g/mL. A high bulk density number implies that there is less occluded air inside the powders, which reduces the probability of product oxidation and increases storage stability. High bulk density suggests a lesser volume for packaging (Rashwan et al., 2022).

Furthermore, the color properties of food materials or products are the most important quality that impacts the marketability and customer acceptability of food products. Hence, the color of YS could be one of the most obvious attributes of powder quality (Liu, Liu, Fan, Qin, & Wang, 2020; Oliveira et al., 2021). The color properties of YS were described in L* (lightness to darkness), a* (redness to greenness), and b* (yellowness and blueness). Moreover, the Hue angle (*H*°) is the ratio of a* and b* and measures the property of color, and the chroma (C*) value indicates the color intensity or saturation. The whiteness index (WI) is a numerical value used to indicate the degree of whiteness of a material, which is calculated using the colorimetric values from the L*, a*, and b* color space, which provides a comprehensive way to quantify color (Rashwan, Karim, et al., 2024) . The color parameters of YS are presented in Fig. 1A**-II**, which have excellent color properties, including L* (92.58 ± 0.05), a* (−1.47 ± 0.27), b* (5.87 ± 0.05), *H*° (−75.95 ± 2.36), C* (6.06 ± 0.11), and WI (90.42 ± 0.04). The color properties indicate that YS is light-colored with low intensity in redness and yellowness, which can be recommended for use in products that require a uniform color, such as yogurt, ice cream, and sweets (Oliveira et al., 2021), where the high whiteness index reinforces this attribute. The values of L*, a*, and b* presented by YS were closer to the values reported by Liu et al. (2020), who found L*, a*, and b* of YS were 96.85 ± 0.07, −1.19 ± 0.28, and 0.98 ± 0.35, respectively.

Additionally, the water solubility (WS%), water absorption index (WAI%), and swelling power (SP g/g) of YS at two temperatures: 80 °C and 95 °C are presented in Fig. 1A**-III**. The modes of swelling and solubility offer insights into the bonding properties within starch granules, where the arrangement and concentration of amylose and amylopectin molecules in the granule structure significantly influence these properties (Ma et al., 2021; Shao et al., 2020). YS showed a higher WS at 95 °C (20.08 ± 0.55) than that at 80 °C (19.77 ± 0.73), where the heat breaks down the starch granules and makes them more soluble. This means that higher temperatures make YS more soluble in water. These results agree with previous findings that YS showed a high WS at higher temperatures, where WS was 17.06 ± 1.13 % at 95 °C compared to 14.50 ± 0.13 % at 85 °C (Liu et al., 2020). Besides, SP at 95 °C is higher than that at 80 °C, reflecting the greater capacity of starch to swell at elevated temperatures. This result is similar to that reported in the study by Shao et al. (2020), who found that the SP of YS (*Dioscorea opposita* Thunb) at 80 °C was 7.80 ± 0.7 % and increased to 11.3 ± 0.1 % at 95 °C. WAI measures the ability of YS to absorb water and swell, where the obtained results showed that WAI is 10.07 ± 0.58 % at 95 °C and decreased to 7.55 ± 0.55 % at 80 °C, indicating that YS absorbs more water and swells more at higher temperatures (Fig. 1A**-III**). These findings are consistent with earlier research that found an increase in temperature causes an increase in WAI of starch extracted from Hawthorn yam (*Dioscorea rotundata*), where WAI was 3.28 ± 0.05 % at 70 °C and 11.1 ± 0.72 % at 80 °C (Pérez et al., 2021).

### 3.2 Thermal properties and structural characterization of yam starch

#### 3.2.1 Morphological, particle size, and AFM image of starch granules

The morphology of starch granules can be utilized to identify the botanical origin of the starch, as the size, distribution, and shape of the granules uniquely influence their functional properties. At three magnifications, 200, 500, and 800×, the shape and size of YS granules were analyzed using an SEM, where the SEM micrograph (Fig. 1B**)** shows that the starch granules isolated from the greater yam tubers (*Dioscorea alata* L.) have irregular oval, trilateral, elliptical, and oblate shapes. The results also showed that the granule surfaces of YS were smooth and free from cracks, with the shape and morphology resembling those typical of most yam species (Li et al., 2024; Liu et al., 2020; Pérez et al., 2021; Shao et al., 2020). The particle diameter of greater YS is 38.90 ± 5.76 μm for the larger diameters, 26.36 ± 3.19 μm for medium diameters, and 13.77 ± 3.15 μm for the smaller diameters, which was consistent with the result of Zou et al. (2020) and Zou, Xu, Zou, and Yang (2021). While YS is typically thought to have tightly packed granules with smooth surfaces, these granules may contain mesopores and micropores that are too small to be detected by standard SEM or may be obstructed by endogenous surface proteins (Li et al., 2024; Zou et al., 2020). For this reason, the nanostructures of the YS granule surfaces were further characterized using AFM. AFM, a high-resolution nanoscale technology with minimal sample preparation and superior 3D imaging capabilities, has been effectively utilized to examine both the internal and external structures of starch granules in conditions close to their native state (Ma et al., 2021).

**Fig. 2:**
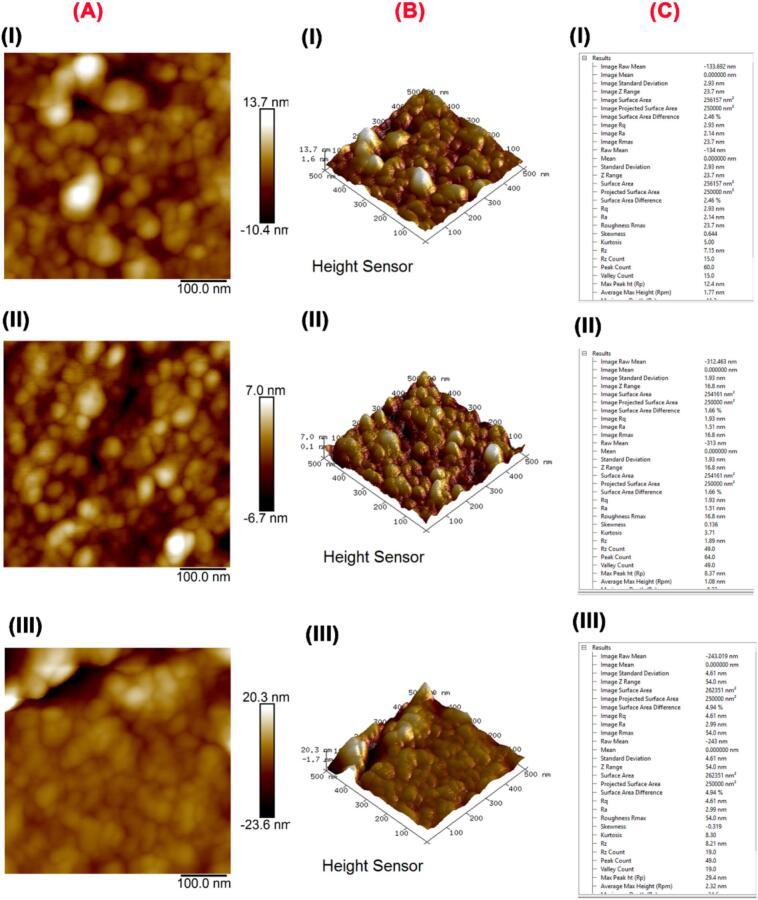
Atomic force microscopy (AFM) scanning of the surface of yam starch granules. (A) Topographical images; (B) 3D surface images (phase images); (C) Quantitative surface analysis.

AFM analysis in Fig. 2 shows significant differences in the surface morphology of starch granules. These differences are important for understanding the performance of materials in various applications. The combination of topographic images, 3D surface images, and quantitative analysis **(Fig. 2AI-AIII)** demonstrates the value of AFM in materials by providing a good overview of the remaining properties. **Fig. 2AI** showed a topographical image of YS with a height variation range between −10.4 nm and 13.7 nm, as well as showing a relatively smooth surface with few distinct features. The presence of bright spots indicates higher regions, suggesting some granule surface irregularities. The second image in **Fig. 2AII** shows a better view with better details. The height change is less pronounced (−6.7 nm to 7.0 nm), indicating a larger difference compared to the image in **Fig. 2AI**. Besides, **Fig. 2AIII** exhibits the most significant height variation (−23.6 nm to 20.3 nm), indicating a highly irregular surface with prominent peaks and valleys. This suggests a rougher texture and more pronounced surface features. This change may be due to differences in the function or properties of starch granules (Oliveira et al., 2021; Shao et al., 2020). Additionally, Fig. 2B provides a three-dimensional view of the starch granule surface, showing the different stages. The first 3D surface image is consistent with the field data and shows the center of the irregularity. The peaks and valleys can be seen but not very clearly; the surface is smooth (**Fig. 2BI**). The second 3D surface image in **Fig. 2BII** shows a detailed surface with a higher density of small particles. Compared to the image in **Fig. 2BI**, the texture looks more consistent with less variation in height. In addition, **Fig. 2BIII** clearly shows the roughness containing large peaks and deep valleys, consistent with the ground image. This means the surface is uneven and textured (Li et al., 2024; Ma et al., 2021; Oliveira et al., 2021). Furthermore, the quantitative surface analysis data presented in Fig. 2C provide numerical data for various surface parameters. Surface roughness of starch granules can influence their functional properties, such as gelatinization, enzymatic degradation, and interaction with other substances. For instance, a rougher surface might enhance the granules' ability to bind with other molecules, affecting their performance in food or pharmaceutical applications (Pérez et al., 2021).

#### 3.2.2 Fourier-transform infrared spectroscopy (FT-IR)

**Fig. 3:**
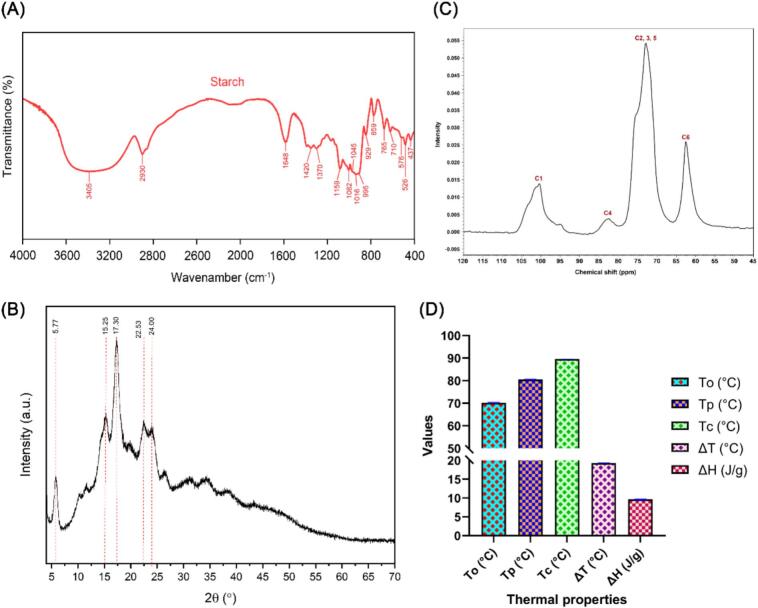
**(A)** Fourier-transform infrared spectroscopy (FT-IR); **(B)** X-ray diffraction (XRD); **(C)** Nuclear magnetic resonance (NMR) spectroscopy; **(D)** Differential scanning calorimetry (DSC) analysis of yam starch. Here, the onset temperature (*T*_o_), peak temperature (*T*_p_), conclusion temperature (*T*_c_), the difference between *T*_c_ and *T*_o_ (*ΔT*), and crystal melting enthalpy (*ΔH*).

The infrared spectrum of YS (*D. alata* L.) in the 4000–400 cm^−1^ area revealed the presence of various functional groups by the creation of bands and peaks (Fig. 3A). These bands and peaks are created by the vibrations of the bonds present in the amylose and amylopectin molecules (Oliveira et al., 2021). The broad band at 3405 cm^−1^ is indicative of O—H stretching vibrations, which are attributed to the hydroxyl groups within the starch molecules, suggesting extensive hydrogen bonding, which is a common feature in polysaccharides such as starch (Li et al., 2024). The intensity and broadness of this band are influenced by moisture content and the extent of hydrogen bonding. The peak at 2930 cm^−1^ corresponds to C—H stretching vibrations, associated with the methylene (CH_2_) groups in the glucose units, indicating the aliphatic nature of the starch structure (Fang, Fowler, Sayers, & Williams, 2004). The band at 1648 cm^−1^, caused by bending vibrations of water molecules (H-O-H), demonstrates starch's hydrophilic nature and capacity to absorb and hold water. Moreover, the bands at 1420 cm^−1^ and 1370 cm^−1^ correspond to CH_2_ group bending vibrations, indicating the presence of methylene groups (Li et al., 2004; Zou et al., 2021). The peaks at 1159 cm^−1^, 1082 cm^−1^, and 1016 cm^−1^ are related to C—O and C—C stretching vibrations, indicative of the glycosidic bonds (C-O-C) that bind the glucose units in starch. Furthermore, the band observed at 1045 cm^−1^ corresponds to the ordered structure of starch granules, while the band at 1022 cm^−1^ is linked to the amorphous structure. The absorbance ratio of 1045/1022 cm^−1^ (IR1) serves as an indicator of the degree of order in the starch. Conversely, the absorbance ratio of 1022/995 cm^−1^ (IR2) represents the proportion of amorphous to ordered carbohydrate structures in the outer region of the starch (Jiang et al., 2023; Liu et al., 2020; Lu, Liu, Yu, & Wang, 2022). The IR1 and IR2 of *D. alata* L. starch were 1.12 and 0.98, which corresponded with the findings of Li et al. (2024). Additionally, bands at 929–765 cm^−1^ are attributed to the vibrations of the pyranose ring in the glucose units, offering insights into the conformation of glucose rings (Fang et al., 2004).

#### 3.2.3 XRD and NMR spectroscopy

XRD has been extensively utilized to uncover the crystalline structure characteristics of starch granules, where the external chains of amylopectin can interact with each other and with water, resulting in the formation of a crystalline structure. The internal crystalline organization of starch can be categorized into A-, B-, and C-types based on their unique characteristic peaks, whereas amylose contributes to the development of an amorphous region (Jiang et al., 2023). The XRD spectrum of greater YS (*D. alata* L.) presented in Fig. 3B showed the strongest five intense diffraction peaks at approximately 5.77°, 15.25°, 17.30°, 22.53°, and 24.00° 2θ, as well as weak diffraction peaks not indicated on the spectrum (11.59°, 20.0°, and 26.29° (2θ)). These peaks were characterized as the B-type pattern, which agrees with the results reported by other authors for *D. alata* starch (Li et al., 2018). Besides, other studies showed that *D. alata* L. starch had four strong diffraction peaks at around 5.6°-5.8°, 17°, 22°, and 24° (2θ), indicating that its starch contained mainly B-type crystalline polymorph (Jiang et al., 2023; Lu et al., 2022). Furthermore, the relative crystallinity of starch affects its digestibility, thermal properties, stability properties, and retrogradation behavior, making it an important food product, pharmaceutical, and commercial product. Understanding and controlling starch crystallinity can improve product attributes such as texture, stability, and shelf life. The relative crystallinity of *D. alata* L. starch is 35.82 %, which is closer to the results found by Li et al. (2024) and higher than that found by Jiang et al. (2023). Amylose molecules with large branches are associated with the amorphous region of the granules, while amylopectin molecules with short branches are associated with the crystalline region. In addition to the diluting effect of amylose, amylopectin content and branch chain length also influence the crystal structure (Li et al., 2024; Shao et al., 2020; Zou et al., 2020). Besides, the higher amylopectin content in the starch of *D. alata* L. (Fig. 1A-I) resulted in increased crystallinity, explaining the higher crystallinity of its starch.

Furthermore, the peak position of helix conformations of decomposed peaks from ^13^C CP/MAS NMR spectra of *D. alata* L. starch was examined. The findings presented in Fig. 3C showed that the ^13^C NMR spectra of *D. alata* L. starch can create four signal intensity regions including C_1_ region, C_4_ region, C_2,3,5_ region, and C_6_ region, where C_1_ region ranged from chemical shift ∼94–105 ppm, C_4_ region from chemical shift ∼80–84 ppm, C_2_, C_3_, C_5_ region from chemical shift ∼68–78 ppm, and C_6_ region from chemical shift ∼58–65 ppm (Zou et al., 2020). In the C_1_ region, the B-type helix structure of starch is characterized by distinctive double peaks with chemical shifts at 101 and 100 ppm. In contrast, the V-type helix structure in the C_1_ region is marked by a single peak at 104 ppm (Tan, Flanagan, Halley, Whittaker, & Gidley, 2007). The peak shapes and intensities are more consistent with the broader and less defined features of B-type starch or the intermediate features of C-type starch. These findings were supported by XRD, which revealed crystalline properties (Fig. 3B). Additionally, the crystalline area of starch was predominantly made up of organized double helices (∼67.5 %) in amylopectin side clusters and single helices from the amylose-fat complex. In contrast, the amorphous region (∼32.5 %) was primarily composed of scattered amylose molecules and branched amylopectin molecules (Zou et al., 2020). Moreover, **Fig. S2** shows the ^1^H NMR spectrum of *D. alata* L. starch, with a prominent peak at ∼5.7 ppm and an intensity reaching ∼90 units, which probably corresponds to the anomeric hydrogen atoms in the glucose units of the starch polymer (Tizzotti, Sweedman, Tang, Schaefer, & Gilbert, 2011). The chemical shift of the anomeric hydrogen typically ranges between 4.5 and 5.5 ppm, with the slightly higher shift here possibly due to the solid-state nature of the sample. The baseline shows some noise, with negative intensity values reaching approximately −15 units, possibly due to instrumental factors or sample preparation issues (Wang, Ma, Gao, Wang and Zhang, 2022). Moreover, the chemical environment in solid samples differs from that in solution samples, with hydrogen atoms in crystalline regions showing different chemical shifts compared to those in amorphous regions.

#### 3.2.4 DSC and degree of gelatinization

Starch gelatinization is the process of the crystalline starch granules losing their granular integrity, melting crystalline areas, and separating and unfolding of double helices in the crystal register when the starch-water combination is heated (Shao et al., 2020; Sorour et al., 2019). The transition temperatures, which are influenced by granule size, the amylose-amylopectin ratio, and the kind of crystalline organization, correlate to changes in the swelling, hydration, and rupture of the structure of YS (Oliveira et al., 2021). The starch gelatinization temperature and enthalpy, which reflected the energy needed to break down the starch crystal, were determined using differential scanning calorimetry (DSC) and starch dyed with Congo red dye (0.2 %). The gelatinization temperatures (*T*_o_, *T*_p_, and *T*_c_), temperature range *△T* (*T*_c_ – *T*_o_), and enthalpies of gelatinization (*△H*_gel_) are presented in Fig. 3D. *T*_o_, *T*_p_, *T*_c_, *△T °*C, and *△H*_gel_ (J/g) of *D. alata* L. starch is 70.22 ± 0.03, 80.53 ± 0.02, 89.53 ± 0.04, 19.31 ± 0.03, and 9.66 ± 0.03 °C, respectively. These findings are also supported by the results obtained from **Fig. S3,** which presented the starch dyed with Congo red. The Congo red dye test was used to discriminate between native, gelatinized, and damaged starch, where *T*_o_, *T*_p,_ and *T*_c_ are 77, 82, and 85 °C, respectively. The higher *T*_o_, *T*_p_, *T*_c_, *△T*, and *△H* of YS could be attributed to the crystallites with distinct thermal stability in the starch (Lu et al., 2022). This observation aligns with the parameters reported for starches in other tuber crops, such as cassava, potato, and sweet potato (Rashwan, Younis, et al., 2024). Additionally, the current results are consistent with the parameters reported for Dasan YS, where it showed the highest peak temperature for gelatinization, including *T*_o_, *T*_p_, *T*_c_, *△T*, and *△H* (75.7 ± 0.8, 82.4 ± 0.3, 88.3 ± 0.3, 12.6 °C, and 16.7 (J/g), respectively (Huang, Lin, & Wang, 2006). In contrast, the results by Li et al. (2024) reported lower *T*_o_ (65.65 °C), *T*_p_ (69.88 °C), *T*_c_ (74.77 °C), and *△H*_gel_ (10.25 J/g) of Pingliang YS than our results; however, the results of gelatinization temperature and enthalpy for Foushou YS were higher than our results for *D. alata* L. starch.

#### 3.2.5 Yam starch stained with APTS and fluorescamine

**Fig. 4:**
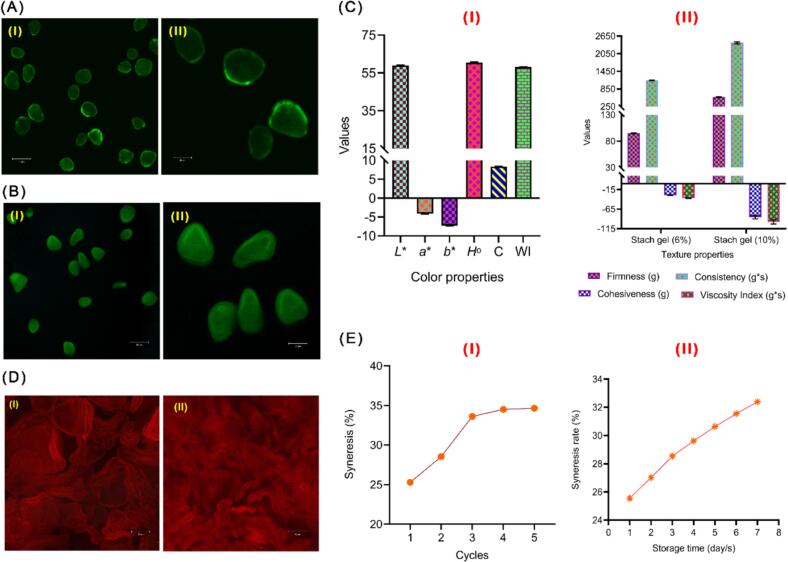
Confocal laser scanning micrographs (CLSM) images of yam starch granules stained with **(A)** fluorescamine and **(B)** 8-Aminopyrene-1,3,6-trisulfonic acid trisodium (APTS). Here, (I): zoom 1.0 and (II): zoom 2.5, **(C—I)** Color properties of yam starch gel (6 %), and **(C-II)** Texture properties of yam starch gel (6 and 10 %), **(D)** CLSM images of the microstructure of yam starch gel (3 % *w*/*v*) stained with Congo Red at (I) 85 °C and (II) 95 °C and then cooled down to room temperature; **(*E*-I)** The freeze-thaw stability of yam starch gel; **(E-II)** Retrogradation properties as syneresis rate (%) of yam starch gel at 1–7 days. The values are expressed as the mean ± standard deviation (SD). **Here**, L*: Lightness, a*: Redness, b*: Yellowness, *H*°: Hue angle, *C*: Chroma, WI: Whiteness index. (For interpretation of the references to color in this figure legend, the reader is referred to the web version of this article.)

CLSM images of staining YS granules with fluorescamine and APTS are shown in Fig. 4A-B, where these dyes are mainly used to study the architecture of the outer shell and inner blocklets of YS granules. Fluorescamine is commonly used to visualize the interior structure of starch granules, which is inherently non-fluorescent; however, it readily combines with primary aliphatic amines to produce highly fluorescent derivatives, whereas unreacted fluorescamine hydrolyzes in seconds to non-fluorescent compounds (Ji, 2020; Ma et al., 2021). Proteins have been found to relate to both the surface and interior of granules, which are known as starch granule-associated proteins (SGAPs) are found in the channeled microstructure of starch granules and are firmly embedded on the granule surface, in granule channels, and in the granule matrix. The existence of SGAPs may serve as the first barrier to processes such as granule hydration, enzyme assault, and chemical interaction with modifying agents (Ma et al., 2021). CLSM is quite effective in detecting the residual protein in YS granules. Staining YS granules with fluorescamine (Fig. 4A) demonstrates the presence and distribution of amine groups within starch granules, implying that protein or amino acids are linked to starch molecules. The uniform fluorescence suggests a homogeneous distribution of these amine-containing compounds. The interior structure of the granules and the distribution of amine groups are more visible at increasing magnification (**Fig. 4AI**). The fluctuation in fluorescence intensity may be a sign of variations in the concentration of amine groups between or within granules (Ma et al., 2021). Additionally, YS stained with APTS from CLSM images is shown in Fig. 4B. APTS selectively reacts with the reducing ends of starch molecules. Fluorescence intensity was positively connected to amylose concentration because the molar ratio of the reducing end in amylose was higher than that of amylopectin (Ma, Xu, et al., 2022). As shown in **Fig. 4BII**, the amylose concentration is lower than the amylopectin concentration; thus, few outer shells carried a little of the brighter inner blocklets that had not escaped. The brightness of the outer shell was significantly lower than that of the inner blocklets, indicating that the amylose content of the outer shell of YS is absent compared to the amylopectin content, as well as less amylose was present in the inner blocklets of YS. Here, both dyes stain the starch granules and produce green fluorescence, but they target different molecular components. Fluorescamine highlights amine groups, indicating protein or amino acid presence, while APTS targets carbohydrate moieties, indicating amylose and amylopectin distribution. Understanding the distribution of proteins/amino acids and amylose/amylopectin within starch granules can inform processing and application decisions. In food applications, the protein content can affect nutritional value and allergenic potential, while amylose and amylopectin distribution can influence texture and stability (Ma, Xu, et al., 2022).

### 3.3 Gel properties of yam starch

Knowing the properties of yam YSG is important to determine its suitable applications in the food industry (e.g., bread, ice cream, yogurt, etc.). For example, color influences consumer appeal, with brightness and whiteness conveying purity and quality. Besides, the texture and stability of YSG are influenced by its microstructure, which has an impact on mouthfeel and product consistency (Oliveira et al., 2021). Freeze-thaw stability (F-TS) is vital for preserving YSG integrity during storage while preventing syneresis and texture degradation (Huang et al., 2024; Oliveira et al., 2021). Retrogradation properties of YSG, such as syneresis rate, can influence the gel's shelf life and quality over time, as water release can degrade texture and appearance (Li et al., 2018). Thus, comprehending the properties of YSG can assist in designing high-quality, consumer-acceptable YS-based products with desirable sensory attributes and a longer shelf life.

#### 3.3.1 Color, texture, and microstructure properties

The color properties of YSG are presented in Fig. 4C-I, where the L*, a*, b*, *H*°, C*, and *WI* are 59 ± 0.09, −4.11 ± 0.05, −7.27 ± 0.04, 60.53 ± 0.27, 8.35 ± 0.05, and 58.16 ± 0.09, respectively. The obtained results indicate that YSG has a moderate light color (L*), the color leans towards green rather than red (a*), the color leans towards blue rather than yellow (b*), the color leans towards the greenish-blue part of the spectrum (*H*°), relatively low color intensity (C*), and a moderate level of whiteness (*WI*). The translucency and light-scattering properties of YSG can be affected by its water content, where the higher moisture content can lead to a more translucent appearance, affecting the L*, a*, and b* values. The texture properties of YSG were carried out after 24 h of cold storage of two starch concentrations in gel, including 6 and 10 % (*w*/*v*). The results of the instrumental texture measurements are presented in Fig. 4C**-II**, where the texture properties measured include firmness (g), consistency (g*s), cohesiveness (g), and viscosity index (g*s). According to the data, the hardness, consistency, and viscosity index of the gel are greatly increased when the starch concentration is raised from 6 % to 10 %. This suggests that the gel network is more resilient and stable at higher concentrations. Besides, cohesiveness is increased in negative value from −29.33 ± 0.5 (g) in 6 % to −85.57 ± 4.2 (g) in 10 %, indicating that the gels may not deform plastically but rather are brittle and easily fractured under stress (Liu, Chao, Yu, Copeland, & Wang, 2021). However, these factors, in turn, have been reported to be dependent on not only the starch concentration in the gel but also on the amylose and the structure of amylopectin (Zhang, Li, Wu, Yang, & Ouyang, 2019). Another study showed that starches with hard gels also tended to feature a higher average degree of polymerization and proportion of long chains in amylopectin (Park, Ibáñez, Zhong, & Shoemaker, 2007).

Furthermore, the microstructure of YSG (3 %) at 85 and 95 °C was studied by staining YSG with Congo Red and observed using CLSM as shown in Fig. 4D. Congo Red is a diazo dye that is widely utilized in a variety of scientific applications, including the investigation of starch gel microstructure, where it can attach to amyloid structures and certain starch, making it effective for coloring starch gels. Congo Red binding to starch molecules can improve microscopic picture contrast, making it easier to recognize the gel microstructure, where it can interact with amylose's helical structures as well as amylopectin's branching structure. This interaction can aid in identifying and characterizing the molecules' distribution and orientation within the gel (Lamb & Loy, 2005; Sorour et al., 2019). The CLSM image of the YSG microstructure at 85 °C shows a relatively less dense network structure, in which the starch granules appear swollen and more discrete, indicating partial gelatinization **(**Fig. 4C-I**)**. Besides, the network of YSG microstructure is less interconnected, with visible boundaries between swollen granules. This result suggests that the starch did not fully gelatinize at 85 °C, resulting in a microstructure with granules that retain some of their original form and size. In contrast, the YSG microstructure at 95 °C exhibits a denser and more homogenous network than that at 85 °C **(**Fig. 4C**-II)**. In which the starch granules appear fully gelatinized, with a more continuous gel matrix. Besides, starch granules have lost their boundaries, merging into a more cohesive network. This indicates complete gelatinization and a higher degree of molecular interaction between amylose and amylopectin chains (Li et al., 2024). At higher temperatures, the extent of retrogradation that affects the firmness and stability of the gel is more pronounced because of the complete disruption of the granule structure and subsequent re-association during cooling (Zou et al., 2021). In which the gel structure retains some granule integrity, leading to a less interconnected network at 85 °C. On the contrary, complete gelatinization forms a cohesive and dense matrix, which can have implications for the texture and stability of starch-based food products at 95 °C (Shao et al., 2020). Therefore, apprehending the differences in the microstructure of YSG obtained at varying temperatures can aid in tailoring its textural properties for specific applications in the food industry, such as thickeners, stabilizers, or gelling agents.

#### 3.3.2 Freeze-thaw stability and retrogradation properties

The freeze-thaw stability (F-TS) and retrogradation characteristics of native starches are an important aspect in their utilization as clean-label ingredients in frozen and cooled food products, where it has been demonstrated that syneresis varies greatly depending on the botanical source of starches. Despite being a small part of starch granules, amylose is known to retrograde more quickly than amylopectin; nonetheless, it is unclear how much amylose content contributes to the syneresis of starch gels (Rashwan, Younis, et al., 2024). F-TS of YSG was measured as % syneresis and determined after the 1st, 2nd, 3rd, 4th, and 5th freeze-thaw cycles as presented in Fig. 4E-I. The results showed an increase in syneresis percentage with an increase in the number of freeze-thaw cycles (FTC), reaching 34.66 % after the 5th cycle. This may be due to starch retrogradation accelerating the extent of phase separation as the number of FTC increased. Furthermore, the high setback is related to a high degree of affinity between starch molecules generated by hydrogen bonding (Tortoe et al., 2019). However, YSG exhibited superior freeze-thaw stability, with very little syneresis during the 4th FTC and no water syneresis during the 5th FTC, where the syneresis was 33.62 %, 34.52 %, and 34.66 % during the 3rd, 4th, and 5th FTC, respectively. It could be ascribed to the low amylose content in YS compared to amylopectin content, as shown in Fig. 1A-I, making it less prone to aging and demonstrating high F-TS. Furthermore, the F-TS of starch is related to its chain length distribution, structural features, and phospholipid content, which may explain the increased water syneresis of YSG in the first FTC (Tortoe et al., 2019).

Moreover, Fig. 4E**-II** shows the retrogradation characteristics of YSG as a percentage of syneresis rate (%). The syneresis rate (%) increases steadily from the first day (25.56 % ± 0.06) to the seventh day (32.39 % ± 0.15). This continuous increase suggests that the retrogradation process in YSG is progressive throughout the storage period. However, the difference in syneresis rate significantly decreased throughout the storage period, where this increase was 1.47 % on the 2nd day and only 0.84 % on the 7th day. This may be attributed to the recrystallization and reassociation of the starch molecules (amylopectin) that prevent water from leaving during the cold storage period (Oliveira et al., 2021). According to these findings, the YS exhibited medium to high syneresis based on the storage day. These results agree with the findings from the previous study by Otegbayo, Oguniyan, and Akinwumi (2014) for the syneresis rate of *D. alata* starch gel. Besides, YSG (*D. alata* L.) has a smaller syneresis than YSG (*D. opposita* Thunb) (39.60 %) and cassava starch gel (*Manihot esculenta* Crantz) (42.36 %) (Zou, Li, Wang, Su, & Li, 2023).

### 3.4 Characterization of set-type yogurt (STY) fortified with yam starch

#### 3.4.1 Color, pH, and titratable acidity of STY

Changes in the color parameters, such as L*, a*, b*, *H*°, C*, WI, and *ΔE* of all yogurt samples during cold storage are shown in Table 1. Color property is considered one of the important quality parameters, which can indicate product stability. Color property significantly affects the marketability and consumer acceptability of food products. The results showed that all treatments exhibit white and bright color, where L* was approximately equal to 90. However, STY fortified with 1.5 % YS has a low L* value compared to other formulations in all storage days. This may be due to the color properties of YSG, as shown in Fig. 4C-I, as well as this reduction could be due to the natural pigments in the YS or due to Maillard reactions during the preparation of STY, affecting the color (Rashwan, Karim, et al., 2024). Moreover, all STY formulations exhibit a negative value of *a*∗ and a positive value of *b*∗, indicating green-yellow color characteristics. Nonetheless, STY-control displays constant a* and b* values compared to STY enriched with YS, which show a few minor differences throughout all storage times. Higher starch concentrations result in a drop in a* values while an increase in b* values, particularly in samples containing 1.0 % and 1.5 % starch. The color variations in a* and b* could be a result of the components of YS interacting with the yogurt matrix, affecting the color (Rashwan, Karim, et al., 2024). Besides, the changes in the *H*° values indicate changes in hue, reflecting the overall perception of color. In comparison to the starch-fortified STYs, the STY-control shows more negative *H*° values, indicating a somewhat more greenish tint. In contrast, the addition of starch to STY led to a decrease in the negative values of *H*°, suggesting a change towards a more yellowish color, and this effect becomes more noticeable with increasing YS concentration in STY. C* represents the vividness or intensity of the yogurt color, where STY-control showed low values of C* compared to the starch-fortified STY, which indicates that the addition of YS enhances the color saturation of the yogurt. This effect might be because of the natural color of YS, YSG, or the influence of starch on the microstructure of STY, which can affect color perception (Tortoe et al., 2019). In addition, the values of WI (whiteness) show a general decrease with increasing the concentration of YS in STY. Besides, the ΔE value represents the change in color of STY formulated with different concentrations of YS, including 0.5, 1.0, and 1.5 % compared to the STY-control color. This shows that using YS produces significant changes in the color of STY. However, the color of STYs with added yams is still bright; thus, starch-fortified STY should be perceived as natural and attractive. These findings are consistent with the results of Pérez et al. (2021).

**Table 1:** Physicochemical properties of set-type yogurt (STY)-fortified with various concentrations of yam starch (YS) during storage at 4 ± 2 °C.

Samples	Storage period (days)	Parameters (Mean ± SD)										
		Color parameters							pH	TA (%)	WHC (%)	Syneresis (%)
		L*	a*	b*	***H°***	***C***	***WI***	***ΔE***				
**STY-Control**	1	90.32 ± 0.06^a^	−3.52 ± 0.01^gf^	9.48 ± 0.06^d^	−69.62 ± 0.17^ab^	10.11 ± 0.05^e^	86.01 ± 0.06^a^	0.00	4.54 ± 0.02^a^	0.68 ± 0.01^gi^	82.62 ± 0.47^ef^	17.38 ± 0.47^ef^
	7	90.30 ± 0.04^a^	−3.46 ± 0.05^ef^	9.51 ± 0.25^d^	−69.98 ± 0.22^bcd^	10.12 ± 0.03^e^	85.98 ± 0.03^a^	0.00	4.33 ± 0.00^e^	0.71 ± 0.01^f^	80.54 ± 0.62^hi^	19.46 ± 0.62^bc^
	14	90.26 ± 0.06^a^	−3.51 ± 0.05^fg^	9.59 ± 0.04^d^	−69.90 ± 0.20^abc^	10.22 ± 0.05^e^	85.89 ± 0.02^a^	0.00	3.77 ± 0.00^h^	0.90 ± 0.01^d^	97.10 ± 0.43^j^	20.90 ± 0.43^a^
	21	90.31 ± 0.02^a^	−3.64 ± 0.07^h^	9.58 ± 0.08^d^	−69.19 ± 0.35^a^	10.25 ± 0.09^e^	85.96 ± 0.06^a^	0.00	3.77 ± 0.00^h^	1.05 ± 0.00^c^	79.95 ± 0.61^ij^	20.05 ± 0.61^ab^
**STY + 0.5 % YS**	1	89.79 ± 0.09^b^	−3.39 ± 0.16^de^	9.64 ± 0.10^cd^	−70.60 ± 1.05^cde^	10.22 ± 0.05^e^	85.56 ± 0.09^b^	0.58 ± 0.19^e^	4.50 ± 0.01^b^	0.68 ± 0.00^g^	82.77 ± 0.23^ef^	17.23 ± 0.23^ef^
	7	89.63 ± 0.19^c^	−3.57 ± 0.02^fgh^	9.82 ± 0.05^c^	−70.03 ± 0.15^bcd^	10.44 ± 0.04^d^	85.28 ± 0.14^c^	0.75 ± 0.21^e^	4.33 ± 0.00^e^	0.71 ± 0.01^f^	81.26 ± 0.41^gh^	18.74 ± 0.41^cd^
	14	89.26 ± 0.09^d^	−3.62 ± 0.06^gh^	10.28 ± 0.05^b^	−70.60 ± 0.38^cde^	10.90 ± 0.03^b^	84.70 ± 0.04^de^	1.23 ± 0.02^d^	3.77 ± 0.00^h^	0.90 ± 0.01^d^	82.25 ± 0.74^fg^	17.75 ± 0.74^de^
	21	89.30 ± 0.15^d^	−3.59 ± 0.03^gh^	10.25 ± 0.5^b^	−70.70 ± 0.24^cde^	10.86 ± 0.03^b^	84.75 ± 0.09^de^	1.22 ± 0.13^d^	3.74 ± 0.00^j^	1.06 ± 0.00^b^	83.27 ± 0.95^def^	16.73 ± 0.95^efg^
**STY + 1.0 % YS**	1	89.26 ± 0.05^d^	−3.46 ± 0.10^ef^	10.20 ± 0.03^b^	−71.25 ± 0.56^e^	10.77 ± 0.02^bc^	84.79 ± 0.05^d^	1.29 ± 0.10^d^	4.49 ± 0.01^b^	0.68 ± 0.00^g^	83.43 ± 0.92^cde^	16.57 ± 0.92^fgh^
	7	89.18 ± 0.08^d^	−3.58 ± 0.03^fgh^	10.29 ± 0.04^a^	−70.81 ± 0.15^de^	10.89 ± 0.04^b^	84.65 ± 0.03^de^	1.37 ± 0.04^d^	4.34 ± 0.00^d^	0.72 ± 0.00^ef^	83.50 ± 0.33^cde^	16.50 ± 0.33^fgh^
	14	89.52 ± 0.04^c^	−3.38 ± 0.06^de^	10.65 ± 0.04^a^	−72.41 ± 0.32^f^	11.18 ± 0.02^a^	84.68 ± 0.04^de^	1.31 ± 0.05^d^	3.80 ± 0.00^g^	0.89 ± 0.01^d^	84.29 ± 0.80^bcd^	15.71 ± 0.80^ghi^
	21	89.55 ± 0.10^c^	−3.32 ± 0.02^cd^	10.63 ± 0.05^a^	−72.64 ± 0.10^fg^	11.14 ± 0.05^a^	84.73 ± 0.10^de^	1.34 ± 0.10^d^	3.76 ± 0.00^i^	1.08 ± 0.00^a^	84.90 ± 0.59^ab^	15.10 ± 0.59^ij^
**STY + 1.5 % YS**	1	88.80 ± 0.05^e^	−3.16 ± 0.05^a^	10.11 ± 0.06^b^	−72.64 ± 0.22^fg^	10.59 ± 0.06^cd^	84.59 ± 0.07^e^	1.69 ± 0.11^c^	4.50 ± 0.00^b^	0.67 ± 0.01^f^	83.46 ± 0.76^cde^	16.54 ± 0.76^fgh^
	7	88.68 ± 0.07^e^	−3.28 ± 0.02^bcd^	10.73 ± 0.14^a^	−72.99 ± 0.25^fgh^	11.22 ± 0.05^a^	84.06 ± 0.09^f^	2.04 ± 0.07^b^	4.42 ± 0.00^c^	0.73 ± 0.01^e^	84.31 ± 0.15^bcd^	15.69 ± 0.15^ghi^
	14	88.37 ± 0.01^f^	−3.22 ± 0.04^abc^	10.77 ± 0.20^a^	−73.37 ± 0.43^gh^	11.24 ± 0.18^a^	83.82 ± 0.13^g^	2.26 ± 0.07^a^	3.85 ± 0.00^f^	0.90 ± 0.01^d^	84.51 ± 0.76^bc^	15.49 ± 0.76^hi^
	21	88.38 ± 0.02^f^	−3.18 ± 0.02^ab^	10.73 ± 0.31^a^	−73.50 ± 0.51^h^	11.19 ± 0.29^a^	83.86 ± 0.21^g^	2.32 ± 0.17^a^	3.77 ± 0.00^h^	1.09 ± 0.00^a^	85.63 ± 0.62^a^	14.37 ± 0.62^j^

The values are expressed as the mean ± standard deviation (SD), and different letters indicate (in the same column) significant differences (*p* < 0.05) (as assessed by Duncan's multiple range test). Here, L*: Lightness; a*: Redness; b*: Yellowness; *H°*: Hue angle; *C*: Chroma; *WI*: Whiteness index; *ΔE*: Color difference; TA: Titratable acidity (as lactic acid); and WHC: Water holding capacity.

Furthermore, the changes in pH and TA of STY samples were evaluated throughout the storage time as presented in Table 1. The results showed that the initial pH values of all STYs, even STY-control, were around 4.5, which is a normal pH value for plain yogurt that generally has a slightly acidic pH. On the other hand, the pH values of all STYs were decreased during the 21-day cold storage period (4). The reduction of pH during storage time may be attributed to the transformation of lactose into lactic acid by the fermentation process and by the activity of LAB in the yogurt during storage times (Rashwan et al., 2022). Besides, the highest reduction in pH occurred between 1 and 7 days of cold storage, while all STY formulations exhibit a similar trend in pH decrease. On each tested day, STY fortified with different concentrations of YS showed slight variations in the pH values. However, these differences are relatively minor, suggesting that adding YS does not significantly impact the pH levels during storage. On the contrary, all STY samples showed an increasing percentage of TA upon the decrease of pH values, as demonstrated in Table 1. The level of titratable acidity in STY samples increases over the storage period, and that reflects the increasing production of acidic compounds such as lactic acid. This rise is modest, with all samples showing a similar upward trend in TA levels. Like pH values, the TA values of all STY samples are largely similar, regardless of YS concentration. Thus, the obtained results suggest that the concentration of YS does not significantly affect the pH and total acidity development in STY during storage; thus, starch can be a suitable ingredient in yogurt without affecting its acidic profile, which is important for yogurt's taste, texture, and shelf life.

#### 3.4.2 Water holding capacity and syneresis of STY

WHC and syneresis are two physical factors that can affect yogurt quality because whey separation on the yogurt surface can shorten the shelf life of yogurt and impair consumer acceptance (Rashwan, Osman, & Chen, 2023). Table 1 illustrates the variations in WHC and syneresis in STY-control and STY-fortified with three YS concentrations (0.5, 1.0, and 1.5 %) during 21 days of storage at 4 ± 2 °C. The WHC of STY-control gradually dropped during the storage period, beginning at 82.62 ± 0.47 % and finishing at 79.95 ± 0.61 %. This trend implies that yogurt's ability to hold water gradually decreases over time, presumably due to natural syneresis and structural changes. STY enriched with 0.5 % YS maintains a steady WHC of 83.27 ± 0.95 % over 21 days of storage under refrigeration conditions. The minor variations are not substantial, demonstrating that 0.5 % YS adequately retains water. Besides, STY-supplemented with 1.0 % YS shows a considerable increase in WHC during storage, commencing at 83.43 ± 0.92 % and reaching 84.9 ± 0.59 % by the conclusion of the time. This rise shows that adding 1.0 % YS improves yogurt's water-holding qualities over time. On the other hand, the syneresis degree of STY-control significantly increased from 17.38 ± 0.47 % on the 1st day to 20.05 ± 0.61 % on the 21st day of storage time. Adding 0.5 % YS to STY causes a progressive decrease in syneresis from 17.23 ± 0.23 % to 16.73 ± 0.95 %, as well as the incorporation of 1.0 % starch in the STY effectively reduces water separation at the end of the storage period (Table 1). The lowest syneresis degree is observed in STY enriched with 1.5 % YS, which is reduced from 16.54 ± 0.76 % on the first day to 13.37 ± 0.62 % on the last day of cold storage (4 ± 2 °C). The increase in WHC and decrease in syneresis could be attributable to a variety of factors. Starch can absorb some water and postpone its separation, while starch molecules (amylose and amylopectin) have great hydrophilicity, allowing them to absorb a large amount of water while improving the stiffness of the protein-gel network (Pang et al., 2019; Rashwan et al., 2023). Furthermore, electrostatic interaction between protein and starch (polysaccharide) can contribute to increased WHC and decreased syneresis of yogurt (Rashwan, Karim et al., 2024). Furthermore, the interactions between protein molecules and starch with multiple hydroxyl and anionic groups might generate a complex with positively charged protein clusters and increased hydrogen bonding in the yogurt (Fan et al., 2023, Rashwan et al., 2024).

Furthermore, increasing total dry matter is associated with higher WHC and increased network stability, perhaps reducing syneresis. Pasteurization reduces whey-release-off rates in samples with high total solids by reducing free water and boosting solids content proportion. Furthermore, after 21 days of storage, the STY with the greatest WHC value had the lowest syneresis value. This is because of the lowering of pH during storage, which caused the release of more serum and exerted a contracting impact on the casein micelle matrix (Du et al., 2023; Pérez et al., 2021; Rashwan et al., 2022). The amylose/amylopectin ratio of 24.6 % to 75.4 % is even more critical to its functional performance in the yogurt. This composition directly explains the starch's excellent gelling and stabilizing properties via a synergistic mechanism. During processing and subsequent cooling, the linear amylose chains leach out of the granules and re-associate into a 3D gel network that acts as the structural backbone that both increases viscosity and traps water, significantly reducing syneresis in yogurt (Wang, Liu and Ai, 2022). The relatively high amylose content is essential for the formation of this strong, continuous network, while the mostly branched structure of the amylopectin component is encapsulated within this network. Because its structure sterically hinders amylose from excessive crystallization, the gel is tender and not too firm or brittle. Additionally, the structure of amylopectin is responsible for binding large amounts of water; thus, the yogurt's water-holding capacity improved dramatically (Gong et al., 2024). Therefore, the specific 1:3 ratio of amylose to amylopectin in this YS creates an ideal balance, yielding a strong yet tender gel structure that effectively stabilizes the yogurt matrix.

#### 3.4.3 Microbiological count

**Fig. 5:**
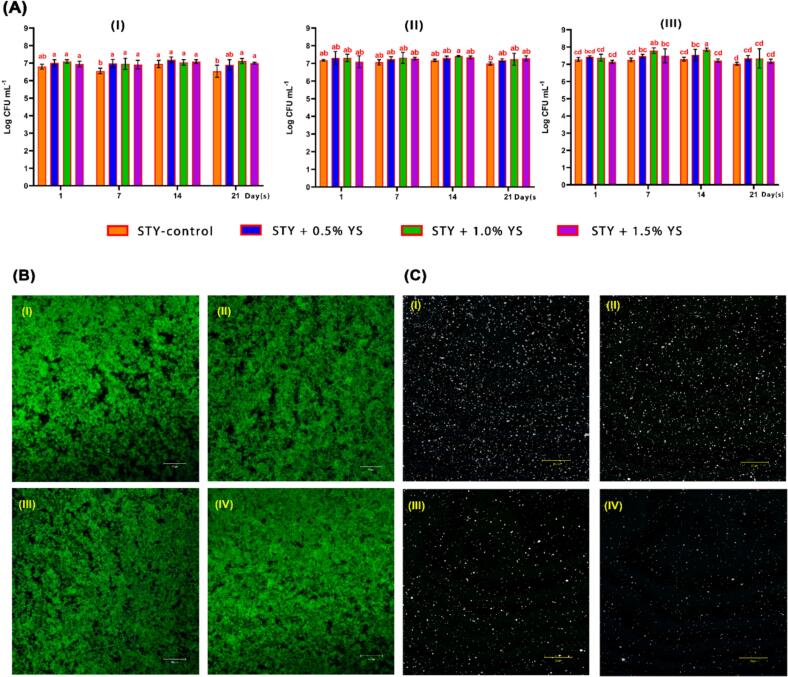
**(A)** Microbiological count analysis (**(I)***Lactobacillus delbrueckii* subsp. Bulgaricus, **(II)***Streptococcus thermophilus*, and **(III)** Total lactic acid bacteria) of set-type yogurt (STY) fortified with different concentrations of yam starch (0, 0.5, 1.0, and 1.5 %) for 21 days of storage at 4 ± 2 °C. The values are expressed as the mean ± standard deviation (SD) and different letters indicate (in the same column color) significant differences (*p* < 0.05) (as assessed by Duncan's multiple range test), (B) microstructures and **(C)** fat globules **(B)** of set-type yogurt (STY) fortified with different concentrations of yam starch after 7 days of storage at 4 ± 2 °C using confocal laser scanning microscopy (CLSM). Here, I: STY-control, II: STY +0.5 % starch, III: STY +1.0 % starch, and IV: STY +1.5 % starch.

Knowing viable bacterial counts of yogurt is important to determine its safety, quality, and nutritional value. *Streptococcus thermophilus* and *L. bulgaricus* are considered the main helpful bacteria that give distinct flavor, texture, and health benefits to yogurt (Rashwan et al., 2023). Yogurt that has been enhanced with starch, like YS, may have an impact on the stability and proliferation of these probiotic bacteria over time. Understanding how starch addition impacts microbial viability is important for preserving the probiotic qualities, shelf life, and consumer attractiveness of the product (Pérez et al., 2021). The viability counts of *S. thermophilus*, *L. bulgaricus*, and total LAB in STY fortified with varying concentrations of YS (0.5, 1.0, and 1.5 %) over a 21-day storage period at 4 ± 2 °C are presented in Fig. 5A. All formulations begin with similar counts of *Lactobacillus delbrueckii* subsp. Bulgaricus ∼7 log CFU/ml (Fig. 5A-I). However, STY-control and STY +1.0 % YS had slightly lower counts than STY +0.5 % YS and STY +1.5 % YS. Despite there being a minor change in the counts over the 21 days of storage at 4 ± 2 °C, the differences between all STY samples are not significant. The STY-control sample has a modest decline on day 7 and then recovers by day 21. However, there are some variations on days 1 and 7, where STY +0.5 % YS has much higher counts than STY-control and STY +1.0 % YS. Additionally, the viability counts of *Streptococcus thermophilus* are ∼7–7.4 log CFU/mL across all STY samples, where the counts remain relatively stable throughout the 21 days of cold storage (4 ± 2 °C) (Fig. 5A**-II**). However, there are minor significant differences on day 1 and day 7, where the STY-control showed a higher count than STY +0.5 % and 1.0 % YS samples. Moreover, viability counts of total LAB for all STY samples ranged from ∼7–7. 7.84 over a 21-day storage period at 4 ± 2 °C (Fig. 5A**-III**). Nonetheless, a notable decrease in the total LAB counts is observed, particularly in the STY-control and STY +0.5 % YS sample, indicating a potential decrease in the viability of these bacteria over time, where these differences are more pronounced in these two samples, especially from day 7 onwards. Overall, STY +1.5 % YS had the highest bacterial counts during the storage period, implying that fortified STY with a high concentration of YS may provide a more favorable environment for bacterial viability, possibly due to the starch acting as a prebiotic substrate or protecting the bacteria from adverse conditions (Pérez et al., 2021; Rashwan et al., 2023). Besides, these values were close to the minimum (6 log CFU/g) required by the Colombian Technical Standard - NTC 805 in yogurts (Pérez et al., 2021).

#### 3.4.4 Texture analysis of STY

The primary indications for assessing the physical and sensory quality of dairy products are the texture parameters, which are influenced by various aspects, including composition and production procedures, and are dictated by the network's structural organization ( Pan, Liu, Luo and Luo, 2019, Rashwan et al., 2024). The results in Table 2 showed that texture parameters, including firmness (g), consistency (g*s), cohesiveness (g), viscosity index (g*s), and gumminess (g) of all STY samples, were significantly increased (*p* < 0.05) within 21 days of cold storage. In comparison with STY-control, STY-fortified YS (0.5, 1.0, and 1.5 %) have higher firmness during the 21 days of storage under refrigerated conditions. Among STY formulations, STY +1.5 % YS showed the highest firmness value, followed by STY +1.0 % YS. This may be explained by the high concentration of starch (amylose and amylopectin) in STY +1.0 % YS and STY +1.5 % YS, where their negatively charged and hydroxyl groups interact with the water molecules through hydrogen and electrostatic bonds to increase their capacity to retain water. Furthermore, hydrogen bonds are highly directed; thus, they can build the water network in the yogurt's structure and strengthen the gel (Rashwan et al., 2024, Rashwan et al., 2024). Furthermore, the protein concentration of the milk base has a direct impact on the consistency of yogurt, with a higher protein content giving yogurt a more solid and uniform structure. Additionally, YS may help stabilize the yogurt gel, which may have a comparable impact on the consistency of the yogurt (Pang et al., 2019). Besides, the supplementation of STY with YS (0.5, 1.0, and 1.5 %) significantly improved (*p* < 0.05) the consistency values than the control during storage at 4 ± 2 °C (Table 2). Among all STY samples, STY +1.5 % YS had better consistency (1658.71 ± 31.47 g*s), followed by STY +1.5 % YS (1515.71 ± 30.80 g*s) compared with STY-control (1205.19 ± 18.85 g*s), especially on the 14th days of cold storage. Cohesiveness is an important parameter to evaluate the yogurt texture and quality, which is a force needed to yank the yogurt stuck to the spoon or mouth upon eating yogurt (Rashwan et al., 2022). In general, water and protein molecules can interact to improve viscosity. On the flip side, yogurt viscosity can also be enhanced because of water interaction with amylose and amylopectin molecules. Because these components have hydroxyl groups, they can interact via hydrogen and electrostatic bonding (Pérez et al., 2021).

**Table 2:** Texture analysis of set-type yogurt fortified with various concentrations of yam starch during storage at 4 ± 2 °C.

Samples	Storage period (days)	Texture parameters (mean ± SD)				
		Firmness (g)	Consistency (g*s)	Cohesiveness (g)	Viscosity index (g*s)	Gumminess (g)
STY-Control	1	37.33 ± 0.44^h^	1134.43 ± 8.20^g^	−24.51 ± 1.10^a^	−32.84 ± 0.87^a^	−915.02 ± 40.35^a^
	7	40.05 ± 2.13^g^	1291.09 ± 45.90^e^	−23.65 ± 2.27^a^	−37.26 ± 1.66^bcd^	−943.99 ± 44.86^a^
	14	39.59 ± 0.55^g^	1205.19 ± 18.85^f^	−23.39 ± 0.29^a^	−35.21 ± 0.14^abc^	−926.41 ± 62.41^a^
	21	37.36 ± 1.04^h^	1147.78 ± 29.26^g^	−22.52 ± 0.73^a^	−33.59 ± 2.10^ab^	−840.83 ± 13.12^a^
STY +0.5 % YS	1	42.05 ± 0.83^efg^	1301.18 ± 48.42^e^	−29.43 ± 1.07^b^	−39.18 ± 1.01^cd^	−1237.32 ± 36.18^b^
	7	43.25 ± 1.77^ef^	1334.52 ± 9.74^e^	−30.06 ± 0.58^b^	−41.09 ± 2.16^de^	−1299.62 ± 42.34^b^
	14	44.30 ± 1.78^e^	1344.14 ± 69.12^f^	−32.77 ± 1.13^c^	−44.12 ± 1.52^e^	−1453.32 ± 107.47^c^
	21	43.66 ± 1.12^ef^	1316.14 ± 19.50^e^	−30.24 ± 1.04^b^	−43.56 ± 2.48^e^	−1319.48 ± 26.01^bc^
STY +1.0 % YS	1	48.19 ± 1.24^d^	1442.33 ± 38.66^d^	−39.02 ± 0.49^d^	−50.10 ± 1.76^f^	−1880.04 ± 43.79^d^
	7	50.64 ± 1.12^c^	1479.00 ± 15.45^cd^	−42.57 ± 1.86^efg^	−52.53 ± 1.55^fg^	−2155.94 ± 118.61^e^
	14	52.58 ± 1.04^c^	1515.71 ± 30.80^c^	−43.44 ± 0.80^fg^	−55.64 ± 3.39^gh^	−2283.26 ± 7.74^e^
	21	51.50 ± 0.93^c^	1502.38 ± 16.90^c^	−41.62 ± 1.37^ef^	−53.28 ± 1.83^gh^	−2142.46 ± 36.24^e^
STY +1.5 % YS	1	61.09 ± 5.05^b^	1582.19 ± 20.02^b^	−40.80 ± 2.21^de^	−55.70 ± 4.32^gh^	−2490.20 ± 80.51^f^
	7	62.06 ± 1.72^ab^	1614.53 ± 20.50^ab^	−44.38 ± 1.48^g^	−61.91 ± 2.85^ij^	−2745.19 ± 108.05^g^
	14	64.19 ± 2.08^a^	1658.71 ± 31.47^a^	−44.95 ± 1.42^g^	−64.61 ± 1.73^j^	−2886.87 ± 179.53^g^
	21	62.96 ± 1.50^ab^	1632.71 ± 43.76^ab^	−43.66 ± 1.67^fg^	−62.95 ± 2.73^j^	−2750.09 ± 169.77^g^

The values are expressed as the mean ± standard deviation (SD), and different letters indicate (in the same column) significant differences (*p* < 0.05) (as assessed by Duncan's multiple range test). Here, STY: Set-type yogurt; YS: Yam starch.

Gumminess, a measure of the energy required to break down yogurt, rises with starch concentration. The control sample has the least gumminess, whereas the STY +1.5 % YS sample has the highest gumminess (Table 2). This rise in gumminess with starch addition could be attributed to the starch's increased stiffness and cohesiveness, which makes the yogurt less susceptible to breakdown. LAB could convert added polysaccharides (starch) to *exo*-polysaccharides, which can assemble with casein proteins, resulting in an improvement in yogurt texture. Similarly, Du et al. (2023) found that adding 0.5 % mulberry pomace polysaccharide to yogurt improved its hardness, consistency, and viscosity index. Besides, another study reported that wolfberry dietary fiber considerably increased the hardness, adhesiveness, gumminess, and chewiness of yogurt (Fan et al., 2023). Furthermore, the significant improvements observed in yogurt's firmness, consistency, and gumminess are a direct manifestation of the yam starch's molecular structure, as elucidated by our XRD and NMR analyses (Fig. 3B and C). The XRD data revealed a relative crystallinity of 32.5 %, indicating a high degree of molecular order. These crystalline regions, which the NMR analysis identifies as organized double helices within amylopectin clusters, act as reinforcing junction zones within the yogurt's gel network (Liu, Chen, et al., 2021). This creates a more robust and interconnected matrix that is more resistant to deformation. Consequently, the increased force required to break down this reinforced structure is measured as higher firmness and gumminess in our textural tests. The stability of these crystallites, evidenced by the gelatinization enthalpy (ΔHgel = 9.66 J/g) from our DSC results (Fig. 3D), further confirms that a more ordered crystalline structure directly contributes to a stronger, more cohesive final gel texture (Scott & Awika, 2023). Therefore, the semi-crystalline nature of the yam starch is the fundamental mechanistic reason for its efficacy as a textural enhancer in the set-type yogurt.

#### 3.4.5 Microstructural and fat globules of STY

Fat globules in milk (yogurt) interact with milk proteins in the yogurt base, where fat globules act as a filler and interact with the protein matrix through fat globule membrane (casein) crosslinks. Thus, fat globules become an integral part of the microstructure of yogurts, reducing fat globules in the whey and thus improving yogurt microstructure (Rashwan et al., 2022, Rashwan et al., 2024). However, fat globules may escape from the yogurt network to the supernatant, rupturing the yogurt network during the centrifugation process, where CLSM was used to investigate the fat globules and microstructures of STY formulations. The results obtained in Fig. 5**-B-C** clearly show the microstructural difference between treated-STYs and STY-control, as well as the fat globules that appeared in the STY samples' supernatants after 7th days of storage at 4 ± 2 °C. The microstructure investigation of yogurts provided a reasonable understanding of the gel formation mechanism and explained the results from other tests, such as physicochemical and textural analysis of both YSG and STY. Adding 0.5, 1.0, and 1.5 % YS to STY significantly (*p* < 0.05) improved the microstructures of STY (**Fig. 5BII-IV**) compared to the STY-control (**Fig. 5BI**). The protein network of STY with YS exhibited fewer pores and channels with much thinner strands of casein aggregates compared to the network of STY-control. The combination of casein in yogurt with starch can generate complexes containing positively charged protein clusters, improving the structure of protein gels. Furthermore, the branch chain structure of starch can promote the covalent cross-linking of casein molecules, resulting in the creation of a larger casein aggregation (Du et al., 2023). Moreover, the current findings revealed that incorporation of various concentrations of YS in STY significantly (*p* < 0.05) reduced the escape of fat globules from the network of yogurt to supernatant after centrifugation compared to STY-control (Fig. 5C). The greater reduction of fat globules release was observed in STY +1.0 % YS and STY +1.5 % YS, respectively, where this reduction might be attributable to the high starch content (amylose and amylopectin), which can interact with fat globules, thereby reducing the escape of fat globules. In which starch molecules contain hydroxyl groups and negatively charged groups that can interact with fat molecules through hydrogen and electrostatic bonds (Fan et al., 2023), which can improve fat retention ability.

Furthermore, Wencheng yam starch's effectiveness as a stabilizer in yogurt can be considered alongside other well-established dairy stabilizers. Specifically, gelatin is the most reliable and powerful hydrocolloid for controlling syneresis, but it is derived from animals, resulting in an undesirable thick texture, which is inadequate for a clean label and is also unsuitable for vegetarians (Arab et al., 2023). Pectin provides a great creamy mouthfeel and is plant-based, but it is expensive and works with a specific level of calcium (Assifaoui, Hayrapetyan, Gallery, & Agoda-Tandjawa, 2024). Modified starches provide better resistance to acid and shear than native starches produced from yam; however, they are labeled “modified starch” on ingredient labels, which is increasingly viewed negatively by consumers because they are searching for natural products (Pang et al., 2019). Native starch from yam represents a compelling alternative, as it provides functional advances in water-holding capacity, texture, and sensory quality, while still maintaining a “clean” label for consumers that prefer unmodified plant-based ingredients (Zou et al., 2020). While native starch from yam may not achieve the ultimate technical performance that gelatins or modified starches can achieve, the data we provided suggest that, when used at the appropriate level (1.0 %), a starch from yam can deliver overall consumer acceptability based on the trade-off between functionality and ingredient positioning. This is particularly valuable in the growing market for natural and clean label yogurt.

#### 3.4.6 Sensory evaluation

The addition of yam starch (YS) reveals a slight positive impact on the sensory attributes of yogurt, including appearance, color, taste, smell, and structure. **Table S1** showed that there is a marginal improvement in appearance scores, ranging from 7.04 in STY-control to 7.2 in STY +1.5 % YS. Color perception also improves, with scores rising from 7.00 (STY-control) to 7.44 (STY +1.0 % YS) before slightly decreasing to 7.4 (STY +1.5 % YS). Taste scores show noticeable improvement from 7.12 (STY-control) to 7.48 (STY +1.5 % YS), indicating a positive contribution to flavor. The smell attribute follows this trend, increasing from 7.08 (STY-control) to 7.44 (STY +1.5 % YS), suggesting an enhancement in aromatic compounds or overall olfactory perception. Structure scores are 7.04 in the STY-control and 7.48 in the STY +1.5 % YS, indicating improved consistency or texture. Acidity perception remains relatively stable, with slight variations from STY-control (7.12) to STY +0.5 % YS (7.28) and stabilizing around 7.24 at higher concentrations. Overall acceptability scores mirror these trends, slightly increasing with YS addition from 7.24 in STY-control to 7.44 in STY +1.0 % YS before slightly declining to 7.32 in STY +1.5 % YS. **Table S1** showed a general decrease in appearance scores observed after 14 days of storage at 4 ± 2 °C, with the STY-control scoring lowest at 6.68, while the STY +1.0 % YS and STY +1.5 % YS scored slightly higher, suggesting that YS may help maintain appearance during storage. Color scores also decline, with STY-control scoring 6.84 and STY +1.5 % YS slightly higher at 7.04, likely due to natural degradation or changes in visual attributes. Taste scores decrease for all samples, indicating a decline in flavor quality, possibly due to starch or component breakdown affecting taste stability. Smell scores also drop, with STY-control at 6.8 and STY +1.5 % YS at 7.16, but YS appears to slightly mitigate this decline. Structure scores for STY-control drop significantly (6.96), while samples with YS show a less pronounced decline, suggesting that YS helps maintain yogurt structure over time. Acidity scores generally decrease, with STY-control scoring the lowest (6.52) and STY +1.5 % YS scoring the highest (6.68), possibly due to changes in the acid-base balance during storage. Overall acceptability follows a similar decline over 14 days, with STY-control scoring lowest (6.8) and STY +1.0 % YS highest (7.16), indicating that YS fortification has positive effects.

## 4 Conclusion

This study successfully demonstrates that native Wencheng yam starch (YS) functions as an effective, clean-label stabilizer for set-type yogurt, addressing a critical need in the modern dairy industry. Through comprehensive characterization, we established that YS possesses an optimal amylose-to-amylopectin ratio (24.6:75.4) and favorable gel-forming properties that translate directly into functional benefits. Systematic evaluation of three YS concentrations (0.5 %, 1.0 %, and 1.5 %) revealed dose-dependent improvements in WHC (up to 84.9 % at 1.5 %), significant reductions in syneresis, and enhanced textural attributes, while maintaining probiotic viability above 7 log CFU/mL throughout 21 days of cold storage. However, sensory evaluation identified 1.0 % YS as the optimal concentration, achieving the highest overall acceptability by balancing physicochemical performance with consumer preference. This highlights the importance of optimizing consumer experience rather than simply maximizing functional properties. We propose that the mechanism underlying these improvements involves the formation of a reinforced 3D network where amylose chains create junction zones that enhance gel strength, while amylopectin's branched structure provides water-binding capacity and prevents excessive rigidity. While this study successfully demonstrates YS performance in STY, its applicability to stirred or fruit yogurts would face challenges due to shear sensitivity and acid instability. Furthermore, industrial-scale implementation will require pilot-plant validation to address challenges related to shear forces during processing, batch-to-batch variability, and long-term economic viability. Future work should include pilot-scale trials to optimize processing conditions, implement low-shear handling practices, establish strict quality control protocols, and explore applications in other fermented dairy products, such as kefir. Despite these challenges, YS offers significant advantages as a cost-effective, plant-based, clean-label stabilizer, making it a promising candidate for commercial yogurt production and broader dairy applications.

## CRediT authorship contribution statement

**Ahmed K. Rashwan:** Writing – review & editing, Writing – original draft, Visualization, Validation, Software, Methodology, Investigation, Formal analysis, Data curation, Conceptualization. **Fanrui Zhou:** Writing – review & editing, Visualization, Methodology, Investigation, Data curation, Conceptualization. **Amged El-Harairy:** Writing – review & editing, Visualization, Resources, Conceptualization. **Wei Chen:** Writing – review & editing, Visualization, Validation, Supervision, Resources, Conceptualization.

## Compliance with ethical standards

Ethical permission was not required for the sensory evaluation test, where the sensory evaluation protocol was designed to be a minimal-risk study, as it involved the tasting of a common food product (yogurt) with a food-grade starch by healthy adult volunteers. In line with institutional guidelines that often exempt such low-risk food consumer acceptance studies from formal ethics committee review. All participants were provided with a full verbal disclosure of the study's purpose, the nature of the samples, and the procedures involved. They were informed that their participation was entirely voluntary and that they could withdraw at any time without penalty. Verbally informed consent was obtained from all participants before their inclusion in the panel. All data collected was anonymized to ensure participant confidentiality.

## Declaration of competing interest

The authors declare that they have no known competing financial interests or personal relationships that could have appeared to influence the work reported in this paper.

## Data Availability

Data will be made available on request.

## Appendix A Supplementary data

Supplementary material Image 1
