# Supplementary material for: Structural, physicochemical, and gel properties of Wencheng yam (Dioscorea alata L.) starch and its application for enhancing set-type yogurt properties
Source: Food Chem X. 2025 Nov 7;32:103215. doi: 10.1016/j.fochx.2025.103215 (PMC12639562; doi:10.1016/j.fochx.2025.103215)
Supplement: Supplementary file 1 — Supplementary material [file mmc1.docx]

**Structural, physicochemical, and gel properties of Wencheng yam (*Dioscorea alata* L.) starch and its application for enhancing set-type yogurt properties**

Ahmed K. Rashwan^1,2,3^, Fanrui Zhou^1,4^, Amged El-Harairy^5,6^*, Wei Chen^1,2^*

^1^ Department of Food Science and Nutrition, College of Biosystems Engineering and Food Science, Zhejiang University, Hangzhou 310058, China.

^2^ Ningbo Innovation Center, Zhejiang University, Ningbo 315100, China.

^3^ Department of Food and Dairy Sciences, Faculty of Agriculture, South Valley University, Qena, 83523, Egypt.

^4^ College of Materials and Chemical Engineering, Southwest Forestry University, Kunming 650224, China.

^5^ Department of Crop and Animal Sciences, Albrecht Daniel Thaer-Institute of Agricultural and Horticultural Sciences, Faculty of Life Sciences, Humboldt-Universität zu Berlin, Albrecht-Thaer-Weg 5, 14195 Berlin, Germany.

^6^ Unit of Entomology, Plant Protection Department, Desert Research Center, 1 Mathaf El-Matariya St., El-Matariya, Cairo 11753, Egypt

* Corresponding authors:

**Dr. Amged El-Harairy, Ph.D.**

Department of Crop and Animal Sciences, Albrecht Daniel Thaer-Institute of Agricultural and Horticultural Sciences, Faculty of Life Sciences, Humboldt-Universität zu Berlin, Albrecht-Thaer-Weg 5, 14195 Berlin, Germany. E-mail: elharaam@hu-berlin.de

**Prof. Wei Chen, Ph.D.**

Department of Food Science and Nutrition, College of Biosystems Engineering and Food Science, Zhejiang University, Hangzhou 310058, China. E-mail: zjuchenwei@zju.edu.cn

| 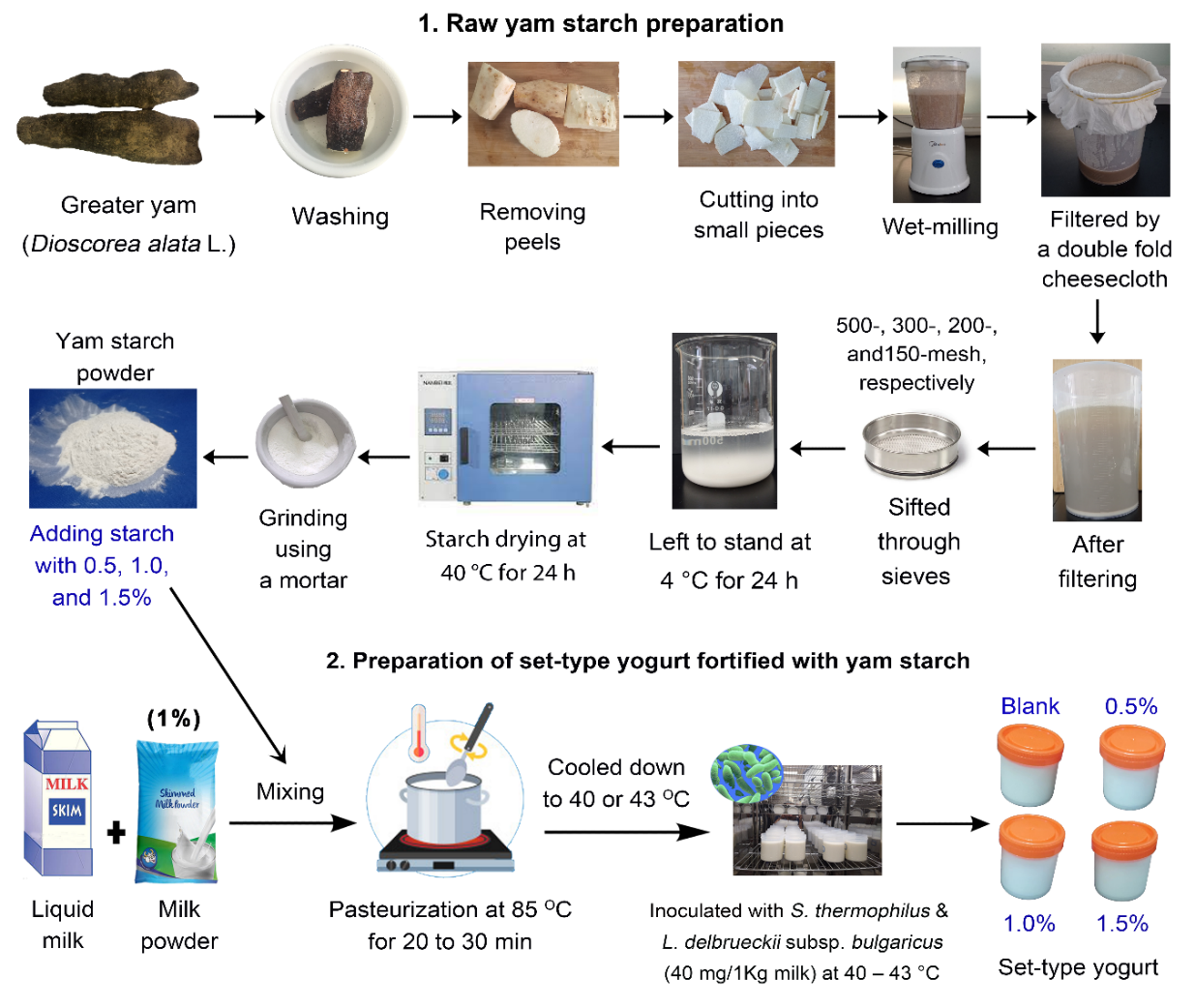 |
| --- |
| **Fig. S1.** The steps of extraction of starch from greater yam tubers (*Dioscorea alata* L.) and preparation of set-type yogurt enriched with different concentrations of yam starch. |

| 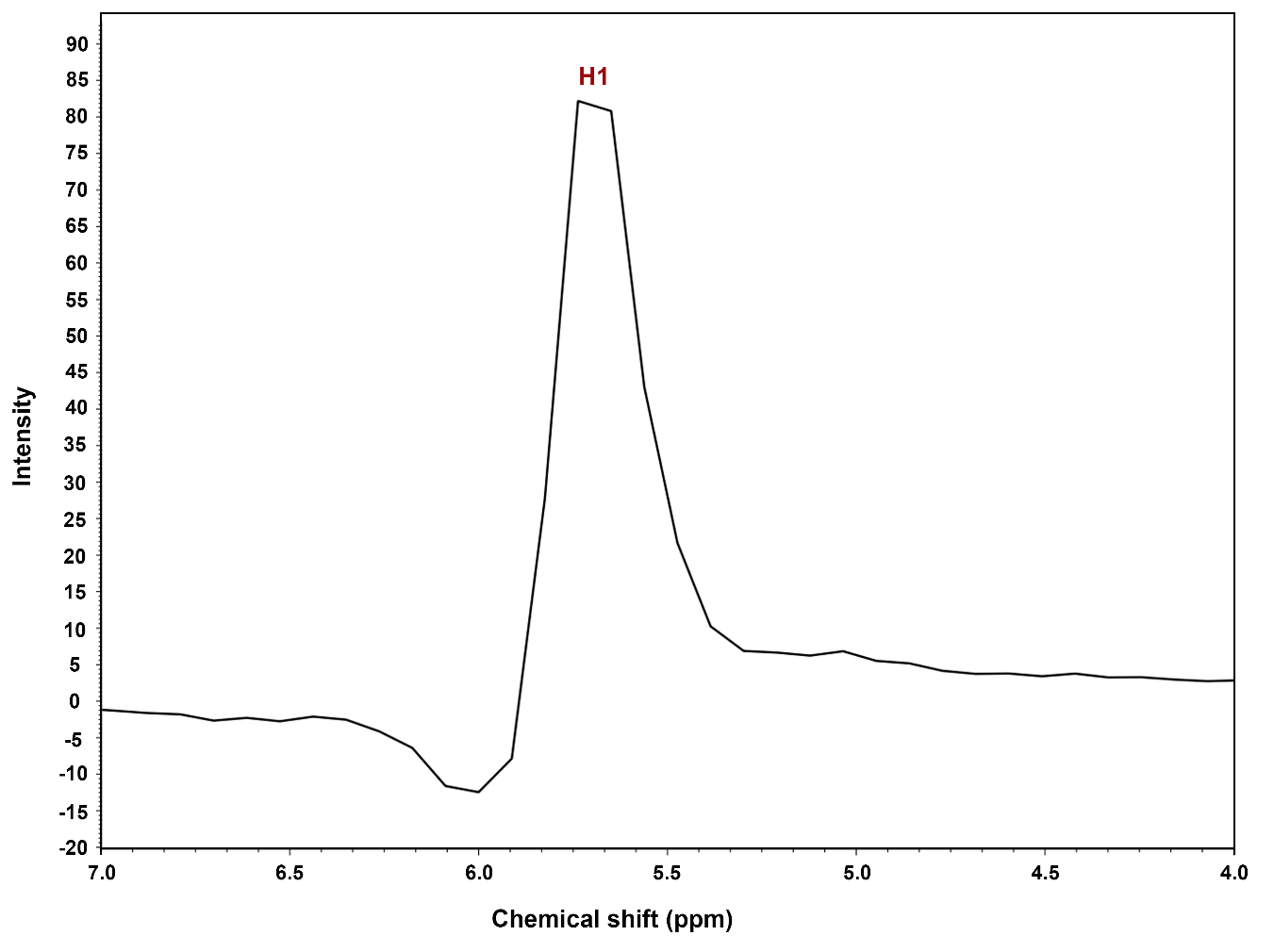 |
| --- |
| **Fig. S2**. ^1^H NMR spectrum of yam starch (*Dioscorea alata* L.) |

## Degree of gelatinization (DG) of YS using Congo red dye

| 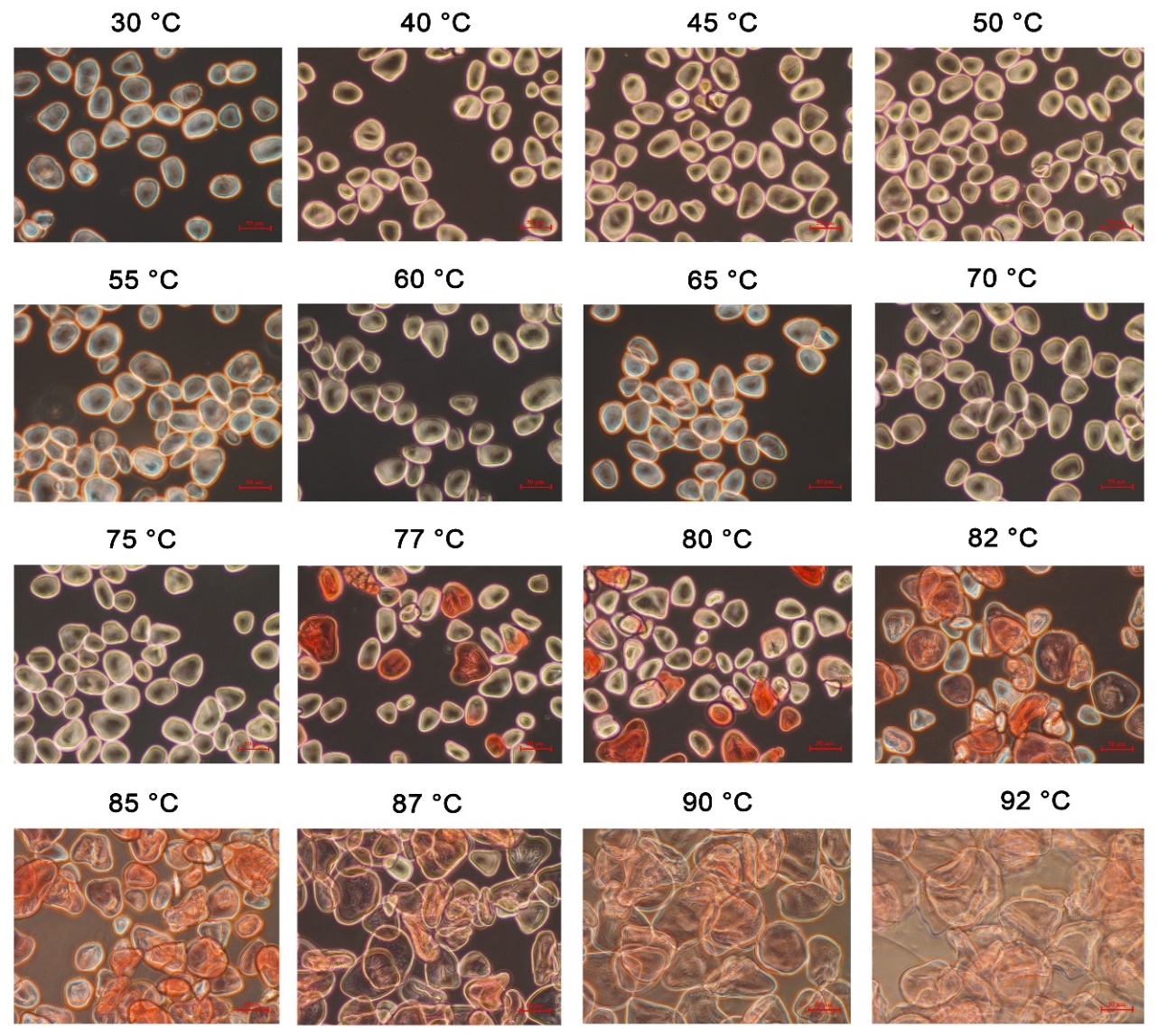 |
| --- |
| **Fig. S3.** Fluorescence microscope images of yam starch granules stained with Congo Red at different temperatures to determine gelatinization temperature range. |

| **Table S1**. Sensory assessment of set-type yogurt (STY) fortified with various concentrations of yam starch (YS) during storage at 4 ± 2 °C. | | | | | | | | |
| --- | --- | --- | --- | --- | --- | --- | --- | --- |
| **Simples** | **Storage period (days)** | **Sensory parameters (mean ± SD)** | | | | | | |
|  |  | **Appearance** | **Color** | **Taste** | **Smell** | **Structure** | **Acidity** | **Acceptability** |
| STY-control | 1 | 7.04±0.73^ab^ | 7.00±0.76^abc^ | 7.12±0.67^abc^ | 7.08±0.76^ab^ | 7.04±0.79^ab^ | 7.12±0.67^a^ | 7.24±0.78^ab^ |
|  | 14 | 6.68±0.069^b^ | 6.84±0.75^c^ | 6.72±0.61^c^ | 6.80±0.58^b^ | 6.96±0.73^b^ | 6.52±0.94^b^ | 6.80±0.50^c^ |
| STY+0.5% YS | 1 | 7.12±0.67^a^ | 7.04±0.73^abc^ | 7.20±0.65^ab^ | 7.12±0.78^ab^ | 7.24±0.72^ab^ | 7.28±0.46^a^ | 7.28±0.74^ab^ |
|  | 14 | 6.84±0.75^ab^ | 6.96±0.73^bc^ | 6.88±0.73^bc^ | 6.92±0.76^b^ | 7.20±0.82^ab^ | 6.36±0.49^b^ | 7.00±0.41^bc^ |
| STY+1.0% YS | 1 | 7.20±0.58^a^ | 7.44±0.65^a^ | 7.36±0.076^a^ | 7.44±0.51^a^ | 7.44±0.82^a^ | 7.24±0.72^a^ | 7.44±0.65^a^ |
|  | 14 | 7.00±0.56^ab^ | 7.00±0.82^abc^ | 7.04±0.73^abc^ | 7.12±0.53^ab^ | 7.28±0.00^ab^ | 6.64±0.70^b^ | 7.16±0.47^ab^ |
| STY+1.5% YS | 1 | 7.20±0.65^a^ | 7.40±0.65^ab^ | 7.48±0.65^a^ | 7.44±0.71^a^ | 7.48±0.77^a^ | 7.24±0.83^a^ | 7.32±0.56^ab^ |
|  | 14 | 7.04±0.73^ab^ | 7.04±0.68^abc^ | 7.20±0.82^ab^ | 7.16±0.69^ab^ | 7.36±0.64^ab^ | 6.68±0.75^b^ | 7.08±0.49^abc^ |
| The values are expressed as the mean ±standard deviation (SD) and different letters indicate significant differences (p < 0.05) (as assessed by Duncan's multiple range test). | | | | | | | | |
